# Community-based psychosocial interventions for people with schizophrenia in low and middle-income countries: systematic review and meta-analysis

**DOI:** 10.1186/s12888-017-1516-7

**Published:** 2017-10-30

**Authors:** Laura Asher, Vikram Patel, Mary J De Silva

**Affiliations:** 1 Centre for Global Mental Health, Department of Population Health, London School of Hygiene and Tropical Medicine, London, UK; 2 Centre for Chronic Conditions and Injuries, Public Health Foundation of India, New Delhi, India; 3 Sangath, Porvorim, Goa India; 4 Department of Global Health and Social Medicine, Harvard Medical School, Boston, MA USA; 5 Wellcome Trust, London, UK

**Keywords:** Schizophrenia, Psychosis, Community mental health services, Psychiatric rehabilitation, Low and middle-income countries, Disability

## Abstract

**Background:**

There is consensus that the treatment of schizophrenia should combine anti-psychotic medication and psychosocial interventions in order to address complex social, economic and health needs. It is recommended that family therapy or support; community-based rehabilitation; and/or self-help and support groups should be provided for people with schizophrenia in low and middle-income countries. The effectiveness of community-based psychosocial interventions in these settings is unclear.

**Methods:**

Studies evaluating community-based psychosocial interventions for people with schizophrenia were identified through database searching up to April 2016. Randomised controlled trials were included if they compared the intervention group with a control group receiving treatment as usual including medication. Only studies set in low and middle-income countries were included. Random effects meta-analyses were performed separately for each intervention type.

**Results:**

Eleven randomised controlled trials in five middle-income countries were identified, with a total of 1580 participants. The content of included interventions varied from single-faceted psychoeducational interventions, to multi-component rehabilitation-focused interventions, to case management interventions. A third of the included studies did not incorporate any community involvement in the intervention. The quality of evidence was often low. Amongst the seven studies that reported on symptom severity up to 18 months post intervention, the pooled standardised mean difference (SMD) across all intervention types was 0.95 (95% CI 0.28, 1.61; *P* 0.005; I ^2^ = 95%; *n* = 862), representing a strong effect. A strong effect on symptom severity remained after excluding two studies with a high risk of bias (SMD 0.80; 95% CI 0.07, 1.53; *P* 0.03; I ^2^ = 94%; *n* = 676). Community-based psychosocial interventions may also have beneficial impacts on functioning (SMD 1.12; 95% CI 0.25, 2.00; *P* 0.01; I ^2^ = 94%; *n* = 511) and reducing hospital readmissions (SMD 0.68; 95% CI 0.27, 1.09; *P* 0.001; I^2^ = 33%; *n* = 167).

**Conclusion:**

The limited evidence from low and middle-income countries supports the feasibility and effectiveness of community-based psychosocial interventions for schizophrenia, even in the absence of community mobilisation. Community-based psychosocial interventions should therefore be provided in these settings as an adjuvant service in addition to facility-based care for people with schizophrenia.

**Electronic supplementary material:**

The online version of this article (10.1186/s12888-017-1516-7) contains supplementary material, which is available to authorized users.

## Background

Provision of anti-psychotic medication alone is inadequate to address the complex social, economic and health needs of those affected by a chronic and highly disabling illness such as schizophrenia. There is therefore consensus that the treatment of schizophrenia should combine anti-psychotic medication and psychosocial interventions [1–4]. Drug treatments generally have most effect on positive symptoms, as well as being effective at preventing relapse [5]. The relative inefficacy of anti-psychotic medication in improving functioning or negative symptoms [6] means a broader supportive approach focused on rehabilitation is also required. Furthermore, the balanced care model proposes that mental health systems should include both community and hospital-based care [7]. Psychosocial interventions typically align with the principles of personal recovery, such as the attainment of a fulfilling and valued life [8].

The Schizophrenia Patient Outcomes Research Team (PORT) evidence-based recommendations, developed in the United States, include eight psychosocial interventions, all of which are recommended as an adjunct to pharmacotherapy: assertive community treatment, supported employment, cognitive behavioural therapy, family-based services, token economy, skills training, and psychosocial interventions for alcohol, substance use disorders and weight management [9]. The strongest evidence is for intensive case management (which has evolved from assertive community treatment) [10], family interventions [11] and psychoeducation [12], with possible impacts on functioning, hospitalisations and relapse rates. However, the quality of evidence is generally low across all types of psychosocial interventions and until recently few studies had been conducted in low and middle-income countries (LMIC). It has also been noted that few of the recommended interventions have been implemented at scale, even in high-income countries [2].

The vast majority of people with mental illness in LMIC do not have access to evidence-based treatments. This is due to chronic underinvestment and a severe shortage of mental health facilities and specialists [13]. Many LMICs are making important strides towards improving care for people with mental illness, in particular through the integration of mental health into primary care [14]. One of five priority Grand Challenges for global mental health is to “Provide effective and affordable community-based care and rehabilitation”, giving recognition to the substantial impact on disease-burden reduction and equity this approach is likely to have, as well as the likely immediacy of impact, and feasibility [15]. However it is broadly accepted that a narrower group of psychosocial interventions for schizophrenia are likely to be feasible in LMIC compared to high-income countries. The third edition of the World Bank’s Disease Control Priorities (DCP-3) recommends that family therapy or support; community-based rehabilitation (CBR); and self-help and support groups should be prioritised in these settings [1]. These interventions may address key challenges to the implementation of psychosocial interventions for schizophrenia in LMIC. First, they may be delivered by non-specialist workers, including nurses without psychiatric training, lay health workers and peer support workers, who are increasingly regarded as the most scalable providers of both pharmacological and psychosocial treatments for schizophrenia in LMIC [16]. This task shifting of mental health care is advocated as a central approach for addressing the shortage of mental health specialists, and ultimately the treatment gap, present in these settings [17]. Second, these interventions may address the broader social and livelihood needs of service users in LMIC. In Ethiopia functional impairment in people with schizophrenia has been conceptualised as arising from severe poverty as much as psychotic symptoms [18]. Some psychosocial programmes in LMIC use an explicit ‘mental health and development’ model focused on economic empowerment [19]. Tailored approaches to mitigate human rights abuses, such as physical restraint, may also be required [20]. CBR places particular emphasis on community involvement, which may entail awareness-raising or mobilisation of practical support from community members. Community mobilisation is seen as the lynch-pin of creating sustainable CBR programmes [21]. Some commentators have cautioned against exporting ‘Western’ diagnoses of mental disorders to settings that have traditionally used alternative explanatory models and labels for distress or disturbed behaviour [22]. It has also been argued that the scale up of biomedical services could marginalize faith and traditional healing [23]. However there have been several successful cultural adaptions of psychosocial interventions across settings and mental disorders [24, 25].

Whilst there is increasing evidence of the acceptability and feasibility of various modalities of community-based psychosocial interventions for schizophrenia in LMIC [26], evidence of effectiveness has been lacking. Given the differences in mental health infrastructure and needs of service users in these settings, a systematic review focused on LMIC is indicated. A systematic review conducted in 2012 by Iemmi et al. identified 15 controlled studies of CBR for a range of physical and mental disabilities. Iemmi et al. reported that overall CBR had a modest positive impact on people with mental disabilities including dementia, schizophrenia and intellectual impairment. However they highlighted the poor quality and non-randomised design of many of the included studies [27]. An initial scoping search undertaken in April 2016 indicated that additional relevant randomised studies had been published since the Iemmi et al. review was conducted. This suggested that an updated review would be of value. Furthermore, the scope of the review was broadened beyond CBR to all community-based psychosocial interventions for schizophrenia. The aim of this review was to assess the effectiveness of all types of community-based psychosocial interventions for people with schizophrenia on patient outcomes in LMIC.

## Methods

### Systematic literature search

#### Eligibility criteria

Individual and cluster randomised controlled trials were included. Eligible interventions were any community-based psychosocial intervention delivered to people with schizophrenia or their caregivers with the aim of improving patient outcomes (see Additional file 1). Studies set in urban and rural locations were included. Psychosocial interventions were defined as any intervention that focused on psychological and/ or social factors rather than biological factors (for example a pharmacological intervention). Interventions could have one or multiple components. Community-based interventions were defined as any intervention delivered in the participant’s home or another community setting. Interventions that took place exclusively in health or other institutional facilities (hospitals, clinics, outpatient care centres or specialised care centres) were excluded. Papers without a full text available in English were excluded due to logistical constraints.

#### Information sources

Database searches were carried out on the 18th and 19th April 2016. The following databases were searched: Medline, EMBASE, PsycINFO, Global Health, CINAHL and Africa Wide information. In addition the Cochrane Library was searched for relevant systematic reviews. The included studies list of each relevant Cochrane review was searched for additional references not already identified in the previous database searches. The Clinicaltrials.gov database was searched for relevant trials; for all trials identified, a search was carried out for relevant linked publications on the clinicaltrials.gov database and on PubMed. The websites of organisations known to conduct relevant research projects and progammes, including BasicNeeds, CBM, and Sangath, were searched for relevant reports and studies. All innovation entries on the Mental Health Innovations Network (MHIN) database were reviewed for relevance and linked publications were sought on PubMed. MHIN is an online platform and database for sharing knowledge, experiences and resources relating to global mental health (www.mhinnovation.net). Reports and guidelines relating to mental health and development or CBR were reviewed for relevant programmes, including the World Health Organisation’s (WHO) CBR guidelines [21], WHO report on Mental Health and Development [28] and the UK government Mental Health for Sustainable Development Report [29]. Relevant literature reviews relating to CBR [27], psychosocial interventions [26, 30], task-sharing [17, 31] and packages of care [1, 2, 32] for mental illness in LMIC were also reviewed for relevant references.

#### Search strategy

The search identified studies covering four domains: A: Schizophrenia or schizoaffective disorder + B: community-based psychosocial intervention + C: low or middle-income country + D: randomised controlled study. Additional file 2 presents the search strategy that was designed for Medline; minor modifications were made as required for other databases. A broad range of search terms were used for domain B, including terms relating to psychoeducation, adherence support, family support, rehabilitation, psychotherapy and counselling, self help groups, health promotion and community-based care. For domain C, separate terms were included for each LMIC, along with generic terms such as ‘developing country’.

#### Study selection

The results of all database searches were downloaded to Endnote X7. Duplicates were removed and the titles and abstracts of the remaining records were screened for relevance. The full texts of those deemed to be relevant were acquired and reviewed. A final list of included eligible studies was compiled after reviewing the full text.

### Data extraction and quality assessment

Key features and findings of each included study were extracted onto a specially designed database. Data were extracted on study characteristics (setting, design, number of participants randomised and duration of follow up), inclusion criteria, characteristics of the interventions (content, frequency and duration) and outcomes. The Cochrane Collaboration risk of bias tool was used to assess each included study [33]. A rating of low, high or unclear risk of bias was given for the following domains: sequence generation; allocation concealment; masking of assessors; selective outcome reporting; incomplete data and other source of bias. Blinding of participants and workers delivering the intervention was not possible due to the nature of the interventions, therefore this criterion was not used.

Statistical analyses were performed using Review Manager 5.3 for Mac. For outcomes measured on continuous scales, the post-treatment mean and standard deviation in the intervention and control groups were extracted along with the sample size in each group. Where these data were presented in the paper, the information was used to calculate the standardised mean difference (SMD) for each trial in order for different outcome scales to be pooled. SMD is a summary statistic that represents the size of the intervention effect in a study relative to the variability observed in that study. The following cut offs were used to guide interpretation of the strength of effect: 0.2 represents a “small” effect, 0.5 represents a “medium” effect, and 0.8 represents a “large” effect [34]. Due to absence of relevant data in the included papers, it was not possible to take into account differences in baseline scores between treatment groups, in the calculation of SMD. Where outcomes were presented as proportions, risk ratios were calculated. For any scale where an increase in score indicates worse outcome, mean scores or proportions were inverted before calculating the SMD or risk ratio. Acknowledging the heterogeneity in interventions, random effects meta-analyses were performed with all intervention types together along with subgroup meta-analyses for each intervention type separately. Meta-analyses were also performed separately for outcomes measured less than 18 months after the intervention ended and outcomes measured more than 18 months after the intervention ended. Heterogeneity between trials was assessed using the I^2^ statistic. In order to understand the impact of study quality on the findings, a sensitivity analysis was conducted excluding studies perceived to have the highest risk of bias overall; these studies comprised those with a risk of bias for allocation concealment, or, for those with an unclear risk of bias for allocation concealment, those studies with a risk of bias for sequence generation or masking of outcome assessment [30]. Lastly, a funnel plot for symptom severity (the outcome utilised by the most studies (*n* = 7)) was generated to assess for publication bias.

## Results

### Overview

**Fig. 1:**
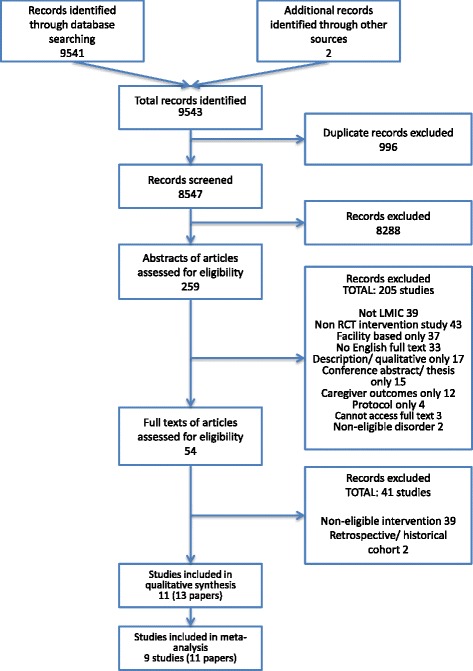
Flow chart of study selection process

From 9543 records, 13 records reporting 11 studies met inclusion criteria for the review (see Fig. 1). The reasons for excluding full text articles are presented in Additional file 3.

### Study characteristics

#### Overview

Table 1 gives a summary of the features of the intervention and study design for each included study. Across all included studies there were a total of 1580 participants with a median sample size of 101, ranging from 45 to 326. Five studies, reported in six papers, were conducted in China [35–40], two studies were conducted in India [41, 42] and Iran [43, 44], one study, reported in two papers, was conducted in South Africa [45, 46], and one study was conducted in Turkey [47]. All studies were conducted in upper-middle income countries apart from the two studies based in India, which is classified by the World Bank as lower-middle income. There were no studies conducted in low-income countries. Five studies took place in urban areas [37, 43–47], two were exclusively in rural areas [35, 36, 38] and one was set across urban and rural sites [41]. The study location was not indicated in three studies [39, 40, 42].

**Table 1:** Summary of the design and findings of included studies

Study and setting	Design and follow up period	Participants [I = intervention C = control]	Intervention duration and content	Personnel delivering intervention	Community involvement	Comparison group	Key results
Group A: Psychoeducation/ cognitive retraining							
Hegde 2012 [42] India^a^	Individual 6 months	Schizophrenia *n* = 45 [*I* = 22, C = 23]	2 months. (i) Cognitive retraining: home visits for cognitive retraining tasks and (ii) Psychoeducation: 3 sessions 45-60 min. Medication.	Researcher	None	Drug treatment and psychoeducation	Symptoms: Positive association with negative symptoms. Cognition: Positive association
Li 2005 [37] China (urban)	Cluster 9 months	Schizophrenia *n* = 101 [*I* = 46, C = 55]	3 months. Family and patient psycho-education in hospital (8 h with patient, 36 h with family) and then at home (2 h/month for 3 months post-discharge). Phases: establish trust, assess needs; psychoeducation, develop coping skills. Medication.	Trained nurse	None	Medication/ standard inpatient care	Symptoms: Positive association at 9 months; no association at 3 months. Functioning: Positive association at 9 months; no association at 3 months. Medication adherence: No association. Knowledge: Positive association
Xiang 1994 [38] China (rural)	Individual multisite 4 months	Schizophrenia and affective psychoses *n* = 77 [*I* = 36, C = 41]	4 months. Family psychoeducation (family visits, workshop, monthly supervision). Medication.	Not stated	Health education through village wired radio network	Monthly drug treatment	Symptoms: Positive association Functioning: Positive association with work ability and poor social functioning. Medication adherence: Positive association
Zhang 1994 [39] China^a^	Individual 18 months	Schizophrenia *n* = 83 [*I* = 39, C = 39]	18 months. Family psychoeducation: initial home visit, then 3 monthly group sessions or individual counseling in outpatients for complex problems; non-attenders had home visits. Minimum contact every 3 months. Medication.	Counsellors	None	Outpatient care - including medication; no active follow up for non- attenders	Symptoms: Positive association Functioning: Positive association Readmission: Positive association Nb All analyses included only those not readmitted.
Group B: Comprehensive family/rehabilitation intervention							
Cai 2015 [40] China^a^	Individual multisite 18 months	Schizophrenia *n* = 256 [*I* = 133, C = 123]	10 weeks. Comprehensive family therapy: (i) Social skills training (medication and symptom management, community re-entry support, recreation for leisure and social independent living skills) 90–120 min/session, 2 sessions/ week for 10 weeks (ii) Family psychoeducation. One session/ week for 10 weeks. Medication.	Professional personnel	None	Usual care (usually monthly outpatient appointment)	Symptoms: No association Cognition: Positive association (greater improvements since baseline compared to control (*p* = 0.002))
Chatterjee 2014 [41] India (urban and rural)	Individual multisite 12 months	Schizophrenia *n* = 282 [*I* = 187, C = 95]	12 months. Collaborative community based care: Home visits fortnightly for 7 months, then monthly for 5 months. Psycho-education; address stigma and discrimination; adherence management strategies; health promotion; rehabilitation strategies to improve social/vocational functioning. Medication.	Lay community health workers	Referrals to community agencies: address social inclusion, access to legal benefits, employment	Facility based care. Psychiatrist consultations. Anti-psychotic medication, information about illness, encouraged medication adherence.	Symptoms: Non-significant association (*p* = 0.08). Functioning: Positive association. Significant differences in PANSS and IDEAS at rural site, but not at others. Medication adherence: Positive association Stigma, knowledge about schizophrenia, caregiver burden: No association.
Ran 2015 [35, 36] China (rural)	Cluster 9 months and 14 years	Schizophrenia *n* = 326 [*I* = 126, C1 = 103, C2 = 97]	9 months. Psycho-educational family intervention (i) Family education 1×/month: information about schizophrenia, relapse prevention, treatment, social functioning rehabilitation (ii) Family workshops 3 monthly (iii) Crisis intervention support. Medication.	Psychiatrists and village doctors	Local village broadcast network used for health education for first 2 months.	1.Medication alone 2. Control (no intervention, medication neither encouraged nor discouraged)	Symptoms: Borderline association 9 months, no association 36 months. Functioning: No association compared to medication alone. Medication adherence: No association compared to medication alone at 9 months. Positive association 14 years. Knowledge: Positive association 9 months.
Group C: Assertive community treatment/ case management/ home after care							
Botha 2014 [45, 46] South Africa (urban)	Individual 12 months and 36 months	Schizophrenia or schizoaffective disorder *n* = 60 [*I* = 34, C = 26]	12 months. Assertive community treatment: individual caseload max 35. Visits >50% at home, fortnightly or according to need. Focused on engagement and maintaining adherence; referral to psychologist, occupational therapist; access to psychosocial rehab program. Medication.	Key worker (social worker or nurse), supported by multi-disciplinary team (psychiatrist, psych nurse)	Strengthening access to existing community resources	Community mental health team: caseload 250+, outpatient appts 1–3 monthly; no active follow up; referral to allied health professionals. Medication.	12 months Symptoms: Positive association Functioning: Positive association Inpatient days & readmissions: Positive association Quality of life and depression: No association 36 months Inpatient days and readmissions: Positive association
Sharifi 2012 [44] Iran (urban)	Individual 12 months	Schizophrenia, schizoaffective disorder, bipolar *n* = 130 [*I* = 66, C = 64]	12 months. Home after care Monthly visits with extra visits in first 3 months. Care plan, drug prescription, dose adjustment, psychoeducation, relapse recognition, referral to hospital. Medication.	General practitioner and social worker- plan reviewed by psychiatrist	Help family to access supportive and community resources.	Hospital outpatient service (no psychosocial component)	Symptoms: Positive association Functioning: No association Readmissions: Positive association Quality of life: No association Depression: Positive association
Ghadiri 2015 [43] Iran (urban)	Individual 20 months	Schizophrenia, schizoaffective and bipolar disorder *n* = 120 [*I* = 60, C = 60]	20 months. Home aftercare (i) Treatment follow up (home visits/telephone and monthly outpatient visit) (ii) Family psychoeducation (six weekly 2h sessions), (iii) social skills training (9 monthly visits). Medication.	Not stated	Contact with local NGOs and self help groups	Usual aftercare including monthly visits by psychiatrist	Symptoms: Positive association Inpatient days and readmissions: Positive association Depression: Positive association
Sungur 2011 [47] Turkey (urban)	Individual 24 months	Schizophrenia *n* = 100 [*I* = 50, C = 50]	24 months. Optimal case management: psychoeducation, adherence strategies, relapse recognition, crisis intervention, family intervention, stress management, social/work skills training. 120 mins every 2 weeks for 3 months at home. Then 45 mins every month at outpatient clinic. Medication.	Psychiatrists, psychologist, psychiatric nurses, supervised by CBT expert.	Referrals to voluntary organisations	Routine case management (outpatient clinic): psychoeducation, adherence support, crisis intervention, day hospital, referrals to rehab. 60 min/month for 3 months then 45 min/month. Medication.	Symptoms: Positive association Functioning: Positive association Quality of life: Positive association Caregiver burden: Positive association

^a^Urban/rural location not specified by study authors

#### Home-based care components

All interventions included a home-based element, a psychoeducation component, and in all studies the intervention group also had access to psychotropic medication. Only the South African study by Botha et al. did not explicitly refer to family involvement in the intervention delivery [45, 46]. Aside from these factors the content and structure of interventions varied between studies. Three broad groups were identified, but with considerable overlap between groups and variation within groups. Group A consisted of largely single-faceted psychoeducation interventions, including three Chinese studies, Li 2005, Xiang 1994 and Zhang 1994 [37–39], and one Indian study, Hegde 2012, that provided cognitive retraining alongside psychoeducation [42]. Group B consisted of more comprehensive multi-faceted interventions including components such as family intervention, support developing social and independent living skills, medication adherence support, crisis intervention and dealing with stigma. This group included the Indian COPSI community-based care trial, Chatterjee 2014 [41], and two Chinese RCTs, Cai 2015 and Ran 2015 [35, 36, 40]. Group C comprised studies focusing on engagement with care following discharge from inpatient facilities, alongside other elements such as social skills training. In this group the South African study, Botha 2014, was based on an assertive community treatment model [45, 46], whilst two Iranian RCTs, Sharifi 2012 and Ghadiri 2015, assessed home-based aftercare services [43, 44], and a Turkish RCT, Sungur 2011, evaluated optimal case management [47]. All Group C studies were based in urban areas.

#### Community involvement components

In five studies, in South Africa, India, Iran and Turkey, individuals were supported to access community resources and organisations including legal benefits, employment opportunities, and non-governmental organisations (NGO) [41, 43–47]. Two Chinese studies, Xiang 1994 and Ran 2015, conducted awareness-raising about mental illness through local radio stations [35, 36, 38]. Four interventions did not include any community engagement or facilitation of support outside of the home-based intervention [37, 39, 40, 42]. Aside from referring to existing community agencies, no studies incorporated active involvement of community members to support individuals with schizophrenia.

#### Personnel

The primary personnel delivering the intervention varied between studies. In three studies, professionals not specialised in mental health, such as social workers or nurses, were the main personnel [37, 44–46]; and in two studies care was delivered by mental health professionals, such as psychiatrists [35, 36, 47]. Only in one study, Chatterjee 2014, was the intervention delivered by lay community health workers [41]. In all but one study the lay community workers and non-mental health professionals worked in a collaborative care model with specialist input [41, 44–46]. In five studies the professional background of the person delivering the intervention was unspecified or unclear [38–40, 42, 43]; the presence of specialist supervision was also not specified in these studies. Interventions were delivered for a median period of 12 months (range 10 weeks to 24 months). The evaluation was conducted immediately on the intervention terminating for six studies and between 6 months to 13 years after the intervention ended for the remaining five.

#### Comparison

Six studies compared the intervention to treatment with medication provision only (typically delivered in an outpatient clinic) and no psychosocial support [37–40, 43, 44]; two studies, Chatterjee 2014 and Hegde 2012, specified that the control included both medication and psychoeducation in an outpatient setting [41, 42]; and the South African and Turkish case management studies, Botha 2014 and Sungur 2011, used outpatient care delivered by a community mental health team as a control [45–47]. One of the Chinese family intervention studies (Ran 2015, Group B) consisted of three arms, comparing (i) a psychoeducational family intervention and medication (ii) medication only and (iii) no intervention and medication neither encouraged or discouraged [35, 36]. In this review only the intervention effects comparing the family intervention and the medication only arm are presented.

#### Outcomes assessed

A wide range of outcomes were assessed including symptoms or clinical state (all studies), functioning (eight studies), medication adherence (four studies), number of hospitalisations (four studies), quality of life (three studies), knowledge about schizophrenia (three studies), depression (two studies), family burden (two studies), cognitive function (two studies), length of hospital stay (two studies), and stigma and discrimination (one study). Clinical symptoms were measured with the Positive and Negative Syndrome Scale (PANSS), the Brief Psychiatric Rating Scale, the mania rating scale and the Current Psychiatric Status-50. Functioning was measured with the Social and Occupational Functioning Assessment Scale, the Social Disability Screening Schedule, the Global Assessment of Functioning, the Global Assessment Scale, the Indian Disability Evaluation Assessment Scale (IDEAS) and ‘working ability’. Depression was measured using the Hamilton Rating Scale for Depression and the Calgary Depression Scale. Quality of life was measured with the WHOQOL (Quality of Life)- BREF and the Quality of Life Scale. Caregiver burden was assessed with the Burden Assessment Schedule and the Scale for the Assessment of Family Distress.

#### Participants and design

There were some differences in diagnoses across studies, with seven studies including only participants with schizophrenia [35–37, 39–42, 47], one study including participants with schizophrenia or schizoaffective disorder [45, 46] and three studies including participants with schizophrenia, schizoaffective disorder or bipolar disorder [38, 43, 44]. Nine studies used an individually randomised design [38–47], whilst two studies used a cluster randomised design [35–37]. Three studies were conducted across multiple sites [38, 40, 41].

### Risk of bias

Overall studies were of low to moderate quality. A summary of the risk of bias for each included study is presented in Additional file 4. Ghadiri 2015, Sharifi 2012, Hegde 2012 and Li 2005 were rated as having a high overall risk of bias [37, 42–44]. Hegde 2012 was excluded from the synthesis of results due to the high risk of bias and the very low sample size included in the outcome analysis (*n* = 12 in treatment group, *n* = 11 in control group) [42]. The findings of Sharifi 2012 are not included in the meta-analysis due to incomplete data presented in the paper (no denominator is given for outcome data). Allocation concealment was adequately described in only one study, whilst procedures were unclear in ten studies. Five studies were assessed to have a high risk of outcome assessors being unblinded, with two studies having a low risk and four studies having an unclear risk. The risk of bias in relation to selective reporting was difficult to assess in seven studies, whilst one study (which had a published protocol [48]) was assessed as low risk and three studies were assessed as high risk (one of which had a published protocol [49]).

### Synthesis of results

#### Symptoms and clinical status

**Fig. 2:**
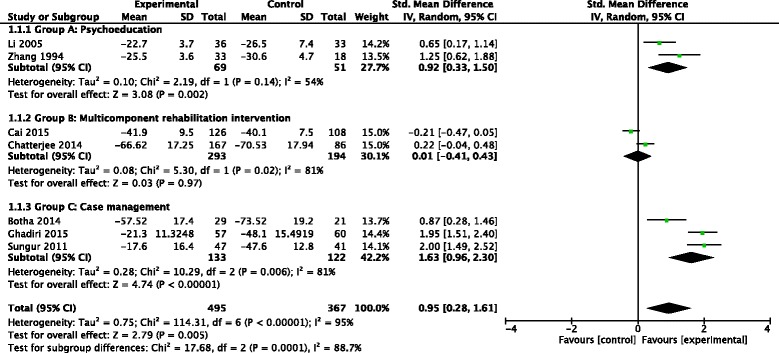
Community-based psychosocial intervention versus usual care: impact on symptom severity (<18 months post intervention)

Amongst the seven studies that reported on symptom severity <18 months post intervention the pooled SMD across all intervention types was 0.95 (95% CI 0.28, 1.61; *P* 0.005; I ^2^ = 95%; *n* = 862), representing a strong effect (see Fig. 2). Excluding the two studies with a high risk of bias reduced the effect size (though this remained ‘strong’), and the precision of the estimate decreased (SMD 0.80 (95% CI 0.07, 1.53; *P* 0.03; I ^2^ = 94%; *n* = 676)) (see Additional file 5).

Ran 2015 also reported on long-term symptom severity, finding no difference between treatment arms at 14 years follow-up [35] (SMD 0.16 (95% CI -0.15, 0.47; *P* 0.3; *n* = 165) comparing the experimental arm and medication control arm). There was some indication that Group B interventions (multi-component rehabilitation interventions) were less effective at reducing symptoms compared to Group A (psychoeducation focused) and Group C (case management) interventions. However this apparent finding should be viewed with caution given the overlaps between intervention type. All four Group C studies (including Sharifi 2012 [44], which was excluded from the meta-analysis due to insufficient data) found a strong association with improvements in symptoms. Whereas, in Group B, Cai 2015 [36] and Chatterjee 2014 [41] did not find a statistically significant difference in symptom severity between treatment arms. There was no clear indication that urban or rural location was associated with a greater impact on symptoms. Whilst all urban-based interventions were effective at reducing symptoms [43–47], Chatterjee et al. found that community-based care led to a reduction in symptoms in rural, but not urban, areas [41].

#### Functioning

**Fig. 3:**
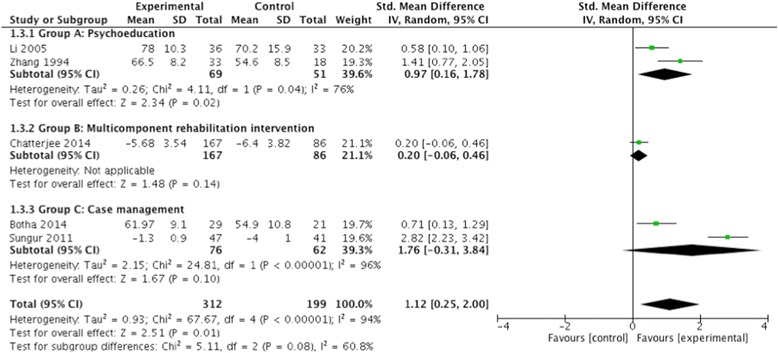
Community-based psychosocial intervention versus usual care: impact on functioning (<18 months post intervention)

Amongst the five studies that assessed functioning <18 months post-intervention using a continuous scale, the pooled SMD across all intervention types was 1.12 (95% CI 0.25, 2.00; *P* 0.01; I ^2^ = 94%; *n* = 511), representing a strong effect (see Fig. 3). All studies in this group were high quality so a sensitivity analysis was not conducted. However, the pooled results of the two studies that measured the proportion able to work <18 months post-intervention did not show an association; the pooled risk ratio was 1.09 (95% CI 0.85, 1.40; *n* = 306) (see Additional file 5). One of these studies, Ran 2015, also measured functioning and work ability after 14 years, but did not find an effect. Comparing the experimental arm and medication control arm, they found an SMD of 0.16 (95% CI – 0.15, 0.47; *P* 0.3; *n* = 165) for functioning and a risk ratio of 1.13 (95% CI 0.93, 1.36) for work ability [35]. Once again the Group B interventions appeared to have the least effect on functioning and work ability. Chatterjee 2014, a Group B study, found a small effect on functioning, though reductions in disability were more prominent in the rural site compared to the two better-resourced urban sites [41].

#### Readmissions and inpatient days

Two Group C (case management) studies, Botha 2014 and Ghadiri 2015, reported on the number of readmissions and number of days in hospital <18 months post intervention. The pooled SMD for number of readmissions was 0.68 (95% CI 0.27, 1.09; *P* 0.001; I^2^ = 33%; *n* = 167) and the pooled SMD for number of days in hospital was 0.55 (95% CI 0.24, 0.86; *P* 0.0006; I^2^ = 0%; *n* = 167), both representing a medium intervention effect (see Additional file 5). The intervention effects remained when Ghadiri 2015, which had a high risk of bias, was excluded. Zhang 1994 (Group A: psychoeducation) also found a positive intervention effect on the proportion with no hospital readmissions over the 18-month period of the intervention (risk ratio 1.83; 95% CI 1.27, 2.64; *n* = 51).

Botha 2014 [45] also reported on outcomes at 2 years after the intervention terminated. They found a strong effect on readmissions (SMD 0.96; 95% CI 0.40, 1.52; *P* 0.0008; *n* = 56) and a medium effect on days in hospital (SMD 0.75; 95% CI 0.20, 1.30; *P* 0.007). It is difficult to determine if Group C (case management) interventions confer any advantage over other types of interventions in reducing readmission rates, as this outcome was not measured for any Group B studies and only one Group A study.

#### Medication adherence

**Fig. 4:**
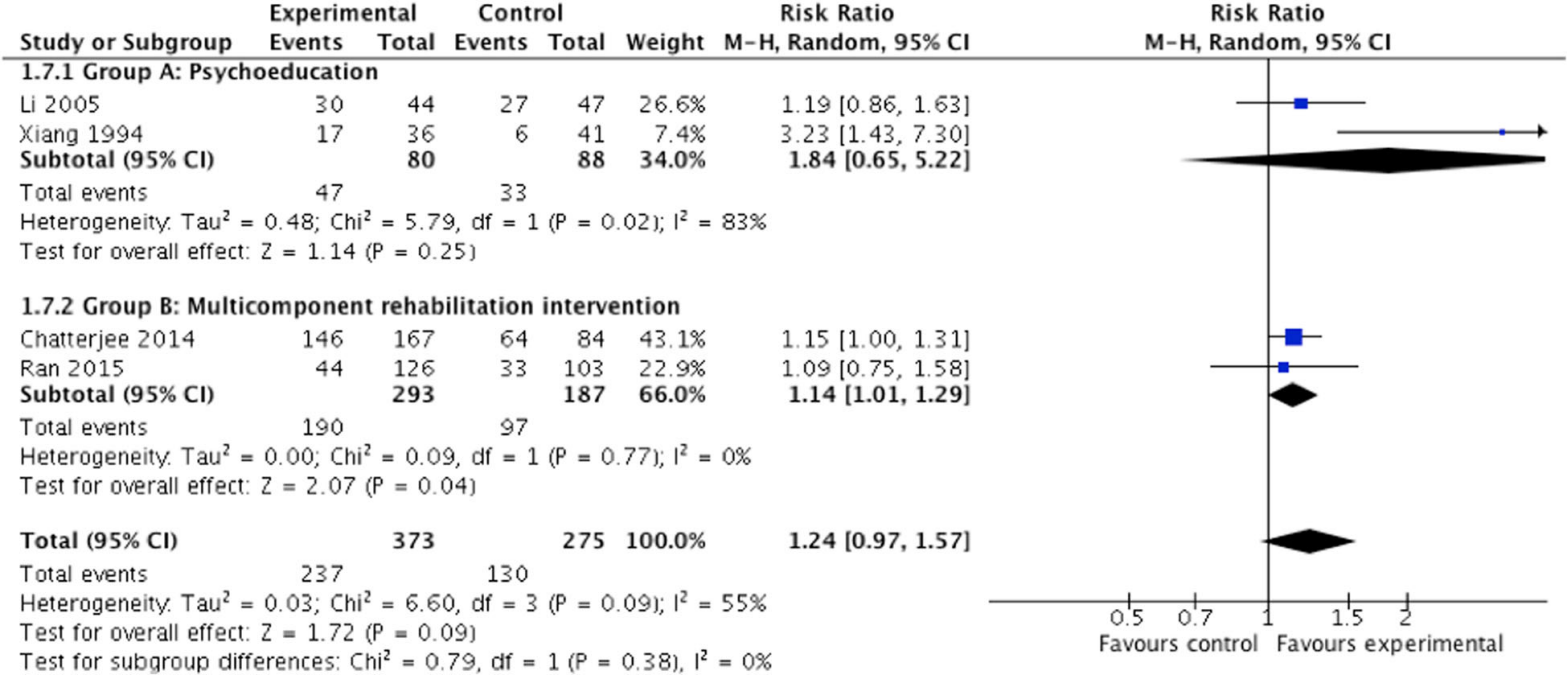
Community-based psychosocial intervention versus usual care: impact on medication adherence (<18 months post intervention)

Two group A (psychoeducation) and two group B (multi-component rehabilitation intervention) studies reported on the proportion of participants who were adherent to medication. There was a borderline significant effect including all studies (risk ratio 1.24; 95% CI 0.97, 1.57; *P* 0.09; I^2^ 55%; *n* = 648) (see Fig. 4). However the precision of this effect was reduced with the exclusion of Li 2005, which had a high risk of bias (risk ratio 1.33; 95% CI 0.90, 1.97; *P* 0.16; I^2^ = 70%; *n* = 557) (see Additional file 5). No studies assessed the long-term impact on medication adherence.

#### Other outcomes

Three Group C (case management) studies reported on quality of life outcomes. Botha 2015 and Sharifi 2012 reported that there was no observed impact on quality of life but did not present the relevant data [44, 46]. However Sungur 2011 found a strong intervention effect on quality of life (SMD 2.05; 95% CI 1.53, 2.57; *P* < 0.001; *n* = 89) [47].

Of the two studies that reported caregiver burden, only Sungur 2011 found an effect (SMD 2.50; 95% CI 1.93, 3.06; *P* < 0.001) [47]. No impact on family burden was observed in Chatterjee 2014 (Group B); insufficient data were provided to calculate the SMD [41].

Two studies, Li 2005 (Group A) and Ran 2015 (Group B), reported significant improvements in knowledge and beliefs about schizophrenia [36, 37]. Li 2005 found a strong intervention effect (SMD 1.04; 95% CI 0.54, 1.55; *P* < 0.001; *n* = 69). Ran 2015 reported favourable differences between treatment arms for six out of eight individual items covering caregiver beliefs and knowledge [36]. No impact on knowledge was observed in Chatterjee 2014 (Group B) (adjusted mean difference 0·34; 95% CI −0·28, 0·96; insufficient data were provided to convert to SMD) [41]. Chatterjee et al. (Group B) were the only study to evaluate the impact on stigma and discrimination; they did not demonstrate an intervention effect [41].

### Publication bias

A funnel plot of symptom severity showed some asymmetry (see Additional file 6). This may indicate that smaller studies without statistically significant effects have not been published.

## Discussion

### Summary of findings

Overall community-based psychosocial interventions in LMICs have a strong effect on symptom severity in people with schizophrenia. There was also evidence of a strong effect on functioning and a medium effect on reducing hospital readmissions, though fewer studies measured these outcomes. These findings were consistent with the 2012 systematic review of CBR by Iemmi et al. [27], but were arguably more robust given the inclusion of eight further RCTs [37, 38, 40–44, 47]. In addition, follow up studies [35, 45] were included of two of the three RCTs relating to schizophrenia that were part of the previous review.

Whilst in some cases there was a substantial impact on outcomes, in other studies such as Chatterjee 2014 the overall impact was slight. However this magnitude of impact may be no different than community-based programmes in high-income countries, and a greater effect was seen in rural areas with fewer resources [41]. There was evidence from only one study, of assertive community treatment in South Africa [45], that positive effects could endure for two years after the intervention terminated. Most studies did not evaluate ongoing effects. Much of the evidence was judged to be of low or unclear quality, meaning conclusions about the effectiveness of these interventions should be made with caution.

The nature of usual care, which differed considerably between studies in this review, should be taken into account when assessing the strength of the evidence. In evaluations where usual care is comprehensive (for example medication, psychoeducation and adherence support offered by psychiatrists in Chatterjee 2014 [41]), smaller gains may be expected from the provision of an adjuvant intervention, compared to evaluations with a low level of usual care (for example medication only in the Chinese psychoeducation-focused RCTs [37–39]). Chatterjee et al. also pointed to the greater impact on disability seen amongst the sub-group who had not previously had access to high-quality facility-based care, compared to those who had [41]. However this supposition does not necessarily hold true; for example Sungur 2011 had large effect sizes yet had one of the most comprehensive packages of usual care (outpatient-based case management) [47].

Several possible mechanisms for the impact of community-based psychosocial interventions present themselves. Supported engagement with treatment and an improved understanding about the nature of the illness and role of medication, by both caregivers and the person with schizophrenia, may lead to improved medication adherence. This in turn may result in improved symptoms and therefore lower relapse rates and fewer hospitalisations. Chatterjee et al. reported a trend towards improved symptoms with improved medication adherence [41], a pattern that has been identified in cohort studies in other LMICs [50]. However only four studies included in this review assessed medication adherence and overall there was a borderline intervention effect. The challenges of intervening to improve medication adherence have been noted across all settings and are not exclusive to mental disorders [51].

It is striking that all types of interventions, including psychoeducation on its own, produced a positive effect on functioning. This may be due to an improvement in symptoms. Other possible pathways to improved functioning are through the impact of improved social skills, improved self-esteem, greater caregiver support, reduced self-stigma or discrimination, or an increased sense of empowerment. However there was almost no assessment of these potential intermediary factors in the included studies. Where the outcomes of quality of life, family burden and perceived stigma were reported, there was less evidence for a beneficial effect of community-based psychosocial interventions.

### Strengths and limitations

Strengths of this study include the inclusive inclusion criteria with respect to intervention content and the robust assessment of study quality. Capturing and synthesising the results of all relevant studies that share the core elements of home-based psychoeducation for schizophrenia in LMICs is a strength of this review. However, the interventions varied considerably in terms of content, intensity, duration and delivery personnel. While the interventions were divided into sub-groups for the meta-analysis, there was variation within groups and overlap between groups.

Whilst the search strategy captured the spectrum of intervention content that may be defined as a community-based psychosocial intervention, rehabilitation programmes based in specialist centres (e.g. [52]) were excluded. This was arguably an unhelpful division, which would not reflect the integrated programming and delivery of psychosocial interventions for schizophrenia in many settings. Outpatient-clinic based psychosocial interventions also represent an important component of services for people with mental illness in LMIC [7]. There is a growing evidence base for such interventions (e.g. [53, 54]) that also requires systematic review. Other methodological limitations of this review include the single screening of records and exclusion of reports not published in English.

### Implications

The results of this review suggest that in LMIC a community-based psychosocial intervention should be provided in addition to facility-based care for people with schizophrenia. Such interventions may have a tangible impact on clinical outcomes. To date there has been limited implementation of psychosocial interventions for schizophrenia in LMIC. The most successful examples of implementation at scale are found in middle-income countries [49, 55]. China’s nationwide ‘686’ programme, which includes active community case finding, community-based care (including multi-disciplinary team input) and hospital care, had achieved 30% coverage of the whole population by 2011 [55]. In low-income countries, whilst community-based psychosocial interventions have been delivered on a small scale by NGOs such as BasicNeeds [19, 56], there are fewer examples of large scale delivery within government run health services [57]. Implementation may vary between low and middle-income settings due to differences in mental health infrastructure and specialists, which are in turn shaped by government prioritization and funding. The included studies from upper middle-income countries tended to take place in the context of well-established inpatient and outpatient mental health facilities [43, 45]. In many low-income countries, mental health care is not available even at the primary care level, let alone at a secondary or tertiary level [58]. The median number of psychiatrists is 0.05 per 100,000 population in low-income countries and 0.54 per 100,000 in lower middle income countries, compared to 2.03 per 100,000 in upper middle income countries and 8.59 per 100,000 in high income countries [13]. This corresponds to a much larger treatment gap for schizophrenia in low-income countries (89%) compared to lower-middle-income (69%) and upper-middle-income countries (63%) [59]. An absence of facility-based care, including provision of anti-psychotic medication, is likely to be a fundamental barrier to providing adjuvant psychosocial support.

Whilst some of the included studies discussed the feasibility and relevance of the intervention for local health systems and other LMICs [41, 45], for many studies it was not clear how or whether interventions could be integrated [37, 40]. Future research should be cognisant of the wider health system, as well as the broader social and economic setting. Most of the included interventions were delivered by health care workers, and in some cases by mental health specialists. This is likely to reflect the upper middle-income setting of nearly all included studies. Even in the COPSI trial, Chatterjee 2014, in which community-based support was delivered by lay health workers, participants received care from psychiatrists in parallel [41]. These interventions, particularly those involving multi-disciplinary teams, may not be feasible in most low-income countries. Moving forward, RCTs of community-based psychosocial interventions are needed in low-income settings, where due to a shortage of human resources the most appropriate personnel are likely to be non-specialist or lay workers [60]. In this review there were no clear indications that interventions delivered by non-mental health specialists resulted in different outcomes compared to those delivered by mental health specialists. This finding strengthens calls for mental health interventions delivered by non-specialists to be prioritised in LMIC, on the basis that they are effective [17] as well as feasible and acceptable [31].

When evaluated in RCTs community-based psychosocial interventions appear to be as effective, or more effective [41], in rural compared to urban settings. However, when implemented at larger scale practical barriers to home-based care delivery may arise, due to the large distances between households and lack of public transport [61]. The need to assess the impact of these feasibility concerns is a compelling rationale for large scale implementation studies.

Another gap in the evidence relates to the scope of interventions. All interventions in this review focused mainly on health issues, with only some touching on social and livelihood elements through skills training. Furthermore there was little emphasis on community mobilisation, beyond the awareness-raising component mentioned in two studies. Where the intervention involved signposting to community resources, there was no detail on whether or how participants accessed these resources. These broader community mobilisation and rehabilitation components form some of the key elements of CBR, which is recommended as an appropriate approach for LMIC. As there is some evidence for the effectiveness of the included studies without these broader components, it is arguable that these elements are not required to achieve improvements in patient outcomes. However it is possible that in low-income settings with few formal health resources, no social security and where the impact of inability to work may be more profound, broader efforts to draw on local community resources and to address livelihood issues may have more relevance.

All included studies assessed symptoms or clinical state, eight studies evaluated the impact on functioning and four assessed mental health service use. Whilst this broadly aligns with outcome measures typically used in similar evaluations in high-income countries [12, 62, 63], recent RCTs have focused on user satisfaction with care [64] and personal recovery [65] as primary outcomes. Personal recovery may also be a pertinent outcome for LMIC countries, however further work is needed to understand the cross-cultural applicability of this concept. To our knowledge only one study, Chatterjee 2014 [41] used a functioning scale specific to the country context. It is proposed that locally adapted functioning scales for psychosis in LMIC offer a more valid measure of disability [66]; such scales should be used wherever possible in future evaluations. A further candidate outcome for LMIC is family-level economic impact, given the important influence of poverty on illness experience in low-income settings [18] and the potential inclusion of livelihood support in psychosocial interventions.

Only three studies assessed outcomes of between 6 months and 13 years after the interventions had terminated [35, 37, 40]. Such study designs, which give valuable information on how to shape psychosocial interventions for scaling up, should be utilised where possible in future research. Of the eleven included studies, only Chatterjee 2014, is known to have conducted in-depth intervention development and piloting in advance of the full evaluation [67]. Formative work is essential to ensure interventions are culturally appropriate and acceptable for the setting, for example by acknowledging local explanatory models or involving faith and traditional healers in the intervention [61]. Chatterjee 2014 also collected process data [41] and conducted a qualitative analysis alongside the trial [68]. For multi-component interventions, theoretical frameworks for the process of change need to be developed to understand which elements contribute towards any impact seen, and why certain elements do or do not contribute to positive effects for participants [69]. Full process evaluations, as well as parallel qualitative studies, are likely to be required. This is particularly pertinent in low-resource settings where low-intensity interventions, employing only the most effective components, may be more feasible for implementation at scale. Alongside a general need for high quality evaluations of community-based psychosocial interventions for schizophrenia, future studies also need to identify and evaluate intermediate outcomes to better understand the mechanisms through which these interventions achieve their impact. Economic evaluations are also needed.

## Conclusion

The limited evidence from low and middle-income countries supports the feasibility and effectiveness of community-based psychosocial interventions for schizophrenia, even in the absence of community mobilisation. Community-based psychosocial interventions should therefore be provided in these settings as an adjuvant service in addition to facility-based care for people with schizophrenia.

## Additional files

Additional file 1: Eligibility criteria. (DOCX 101 kb)

Additional file 2: Medline search strategy. (DOCX 104 kb)

Additional file 3: Reasons for exclusion of full texts. (DOCX 202 kb)

Additional file 4: Summary of risk of bias for included studies. (DOCX 132 kb)

Additional file 5: Additional analyses. This file presents the forest plots for symptom severity (<18 months post intervention) including only high-quality studies; impact on ability to work (<18 months post intervention) including all studies; impact on number of readmissions (<18 months post intervention) including all studies; impact on number of days in hospital (<18 months post intervention); and medication adherence (<18 months post intervention) including only high-quality studies. (DOCX 254 kb)

Additional file 6: Funnel plot for symptom severity. (PDF 65 kb)

## Abbreviations

CBR: Community -based rehabilitation
CI: Confidence interval
DCP-3: Disease Control Priorities, 3rd Edition
LMIC: Low and middle-income countries
MHIN: Mental Health Innovations Network
NGO: Non-governmental organisation
PANSS: Positive and negative syndrome scale
PORT: Patient Outcomes Research Team
SMD: Standardised mean difference
WHO: World Health Organisation
