# Supplementary material for: Community-based psychosocial interventions for people with schizophrenia in low and middle-income countries: systematic review and meta-analysis
Source: BMC Psychiatry. 2017 Oct 30;17:355. doi: 10.1186/s12888-017-1516-7 (PMC5661919; doi:10.1186/s12888-017-1516-7)
Supplement: Supplementary file 1 — Eligibility criteria. (DOCX 101 kb) [file 12888_2017_1516_MOESM1_ESM.docx]

# Additional file 1: Eligibility criteria

|  | **Included** | **Excluded** |
| --- | --- | --- |
| **Publication type** | Any date  English language | No full text available in English. Conference abstracts, Masters dissertations, PhD Theses and unpublished studies. |
| **Study design** | Individual and cluster randomised controlled trials. | Non- randomised controlled intervention studies, case-control or cross-sectional studies. Retrospective/historical controlled cohorts. |
| **Study population** | General adult population | Interventions for children and adolescents (<18 years) |
|  | Study conducted in a LMIC as defined by the World Bank | Study conducted in high-income country or territory |
| **Condition of interest** | Schizophrenia or schizoaffective disorder as defined in International Classification of Diseases-10 or Diagnostic and Statistical Manual of Mental Disorders, 4^th^ Edition confirmed by a clinical diagnosis or a validated tool. No restriction on the proportion of participants with schizophrenia or schizoaffective disorder. No restriction on comorbid mental disorders. | Other mental disorders including depression, substance abuse, bipolar disorder, anxiety disorder, epilepsy, other types of disability, brief psychotic disorders, post-partum/ puerperal psychosis, populations at risk of psychosis. |
| **Intervention** | Community-based psychosocial interventions delivered to people with schizophrenia or their caregivers with the aim of improving patient outcomes. Psychosocial interventions were defined as any intervention that focused on psychological and/ or social factors rather than biological factors (for example a pharmacological intervention), including psychological therapies, rehabilitation, psychoeducation, adherence support, stigma reduction strategies, social skills training, life skills training, self-help groups, group support sessions and livelihoods interventions. Community-based interventions were defined as any intervention delivered in the individual’s home or another community setting. | Studies were excluded if the intervention took place only in health or other institutional facilities (defined as places that provide health care: hospitals, clinics, outpatient care centres, specialised care centres).  The following interventions were also excluded: those specifically designed for humanitarian crisis settings; those employing solely physical therapies including yoga; interventions solely targeting depression, substance use, or smoking; interventions focusing exclusively on ‘at risk’ groups; interventions using telephone reminders only and involving no face to face contact; and interventions which were entirely computer-based (e.g. cognitive training). |
| **Outcome** | Any patient outcomes including  -Clinical outcomes (e.g. symptoms, severity, relapse, mortality)  -Health service use including hospitalisation  -Adherence to antipsychotic medication  -Change in disability and functioning (e.g. improved coping skills, quality of life, social functioning, self-esteem)  -Economic status outcomes, for e.g. return to work, employment status  -Understanding of mental illness/ schizophrenia  -Stigma/ discrimination | Studies were excluded if outcomes were measured in caregivers or family members only |
| **Control Group** | Any control group including treatment as usual, no intervention, treatment delivered in a health care setting or pharmacological intervention only | n/a |
