# Supplementary material for: Community-based psychosocial interventions for people with schizophrenia in low and middle-income countries: systematic review and meta-analysis
Source: BMC Psychiatry. 2017 Oct 30;17:355. doi: 10.1186/s12888-017-1516-7 (PMC5661919; doi:10.1186/s12888-017-1516-7)
Supplement: Supplementary file 2 — Medline search strategy. (DOCX 104 kb) [file 12888_2017_1516_MOESM2_ESM.docx]

## Medline search strategy

Note minor amendments were made to conduct the search in other databases

***A: Schizophrenia***

1. Psychotic disorders/
2. Exp Schizophrenia/
3. Exp Psychotic affective disorders/
4. (Schizophreni* or psychosis or psychoses or psychotic or schizoaffective or Schizophreniform).tw
5. Or/1-4

***B: Community-based psychosocial intervention***

**Psychotherapy**

1. exp Psychotherapy/
2. exp Counseling/
3. Schizophrenic psychology/
4. (Psychotherapy or counselling or counseling or therapy or interpersonal therapy or cognitive behavio?ral therapy or art therapy or music therapy or brief intervention or cognitive retraining or cognitive rehabilitation).tw

**Psycho-education**

1. Health Education/
2. Patient Education as Topic/
3. (psychoeducation* or psycho-education*).tw
4. (Patient* or caregiver* or care-giver* or carer* or family or families) adj3 (education or advice or information or training or support or intervention or livelihood).tw

**Family**

1. Caregivers/
2. Family relations/
3. ((family or families or carer or caregiver or care-giver) adj3 burden).tw

**Adherence**

1. exp Patient Compliance/
2. patient adj3 (compliance or concordance or adherence).tw
3. (antipsychotic* or anti-psychotic* or medication*) adj3 (compliance or concordance or adherence or non-compliance or non-concordance or non-adherence ).tw
4. (Adherence or compliance or medication) adj3 (support or therapy or education or training or advice or information or intervention).tw

**Rehabilitation**

1. exp Rehabilitation/ or Rehabilitation Nursing/ or exp Rehabilitation Centers/
2. Social Adjustment/
3. Cooperative behaviour/
4. Interpersonal relations/
5. Social inclusion.tw
6. (community based rehabilitation or community-based rehabilitation or CBR).tw
7. (rehabilitat* adj3 (home based or home-based or communit*)).tw
8. (Communit* adj3 (vocational training or apprenticeship* or employment placement service* or support network* or self-employ* or supported employ* or social service* or social work*)).tw
9. (Communit* adj3 (personal assistance or personal assistant* or individual support* or disabled people* organi?ation*)).tw
10. (Communit* adj3 (empower* or awareness campaign* or self-advocacy or self-help group* or support group* or women* group* or development group*)).tw
11. (Communit* adj3 inclusi* adj3 (health or education or hous* or social or justice or empower*)).tw
12. Rehabilitation adj3 (vocational or social or personal).tw
13. Training adj3 (Life skill* or social skill* or personal skill* or interpersonal skill* or interpersonal).tw
14. Psychosocial or psycho-social or social or psychological or psychiatric or PSR or vocational or occupational adj3 (intervention* or support or rehabilitation).tw
15. Sustainable livelihood* or livelihood* adj3 (intervention or support).tw
16. Recovery or recovery model or recovery approach or social recovery.tw

**Health promotion**

1. Health promotion/
2. Health adj3 (promotion or advice or information or training or support).tw

**Support group**

1. social support/
2. Self-Help Groups/
3. (Social or peer* or peer-led or peer led or self help or self-help or community or cooperative or co-operative) adj3 (group or support or support group).tw

**Collaborative/ primary/ community-based care**

1. care adj3 (Collaborative or community or community-based).tw
2. (outreach adj3 (service* or care or intervention* or program*)).tw
3. Exp Primary Health Care/
4. Community health services/
5. Home Care Services/
6. Home nursing/
7. Community health nursing/
8. Community networks/ or community mental health services/
9. patient care team/
10. nursing, team/
11. exp social work/
12. community health centers/
13. community mental health centers/
14. community health workers/
15. Outpatients/
16. Ambulatory care facilities/ or outpatient clinics, hospital/
17. Ambulatory care/
18. allied health personnel/
19. nurses aides/
20. psychiatric aides/
21. (nonspecialist* or non-specialist* or allied health or nurse led or community health or village health adj3 (worker* or personnel or team*)).tw
22. or/6-62

***C: Low and middle-income countries***

1. developing countries/
2. (Africa or Asia or Caribbean or West Indies or South America or Latin America or Central America).hw,kf,ti,ab,cp
3. (Afghanistan or Albania or Algeria or Angola or Antigua or Barbuda or Argentina or Armenia or Armenian or Aruba or Azerbaijan or Bahrain or Bangladesh or Barbados or Benin or Byelarus or Byelorussian or Belarus or Belorussian or Belorussia or Belize or Bhutan or Bolivia or Bosnia or Herzegovina or Hercegovina or Botswana or Brazil or Bulgaria or Burkina Faso or Burkina Fasso or Upper Volta or Burundi or Urundi or Cambodia or Khmer Republic or Kampuchea or Cameroon or Cameroons or Cameron or Camerons or Cape Verde or Central African Republic or Chad or Chile or China or Colombia or Comoros or Comoro Islands or Comores or Mayotte or Congo or Zaire or Costa Rica or Cote Ivoire or Ivory Coast or Croatia or Cuba or Cyprus or Czechoslovakia or Czech Republic or Slovakia or Slovak Republic or Djibouti or French Somaliland or Dominica or Dominican Republic or East Timor or East Timur or Timor Leste or Ecuador or Egypt or United Arab Republic or El Salvador or Eritrea or Estonia or Ethiopia or Fiji or Gabon or Gabonese Republic or Gambia or Gaza or Georgia Republic or Georgian Republic or Ghana or Gold Coast or Greece or Grenada or Guatemala or Guinea or Guam or Guiana or Guyana or Haiti or Honduras or Hungary or India or Maldives or Indonesia or Iran or Iraq or Isle of Man or Jamaica or Jordan or Kazakhstan or Kazakh or Kenya or Kiribati or Korea or Kosovo or Kyrgyzstan or Kirghizia or Kyrgyz Republic or Kirghiz or Kirgizstan or Lao PDR or Laos or Latvia or Lebanon or Lesotho or Basutoland or Liberia or Libya or Lithuania or Macedonia or Madagascar or Malagasy Republic or Malaysia or Malaya or Malay or Sabah or Sarawak or Malawi or Nyasaland or Mali or Malta or Marshall Islands or Mauritania or Mauritius or Agalega Islands or Mexico or Micronesia or Middle East or Moldova or Moldovia or Moldovian or Mongolia or Montenegro or Morocco or Ifni or Mozambique or Myanmar or Myanma or Burma or Namibia or Nepal or Netherlands Antilles or New Caledonia or Nicaragua or Niger or Nigeria or Northern Mariana Islands or Oman or Muscat or Pakistan or Palau or Palestine or Panama or Paraguay or Peru or Philippines or Philipines or Phillipines or Phillippines or Poland or Portugal or Puerto Rico or Romania or Rumania or Roumania or Russia or Russian or Rwanda or Ruanda or Saint Kitts or St Kitts or Nevis or Saint Lucia or St Lucia or Saint Vincent or St Vincent or Grenadines or Samoa or Samoan Islands or Navigator Island or Navigator Islands or Sao Tome or Saudi Arabia or Senegal or Serbia or Montenegro or Seychelles or Sierra Leone or Slovenia or Sri Lanka or Ceylon or Solomon Islands or Somalia or Sudan or Suriname or Surinam or Swaziland or Syria or Tajikistan or Tadzhikistan or Tadjikistan or Tadzhik or Tanzania or Thailand or Togo or Togolese Republic or Tonga or Trinidad or Tobago or Tunisia or Turkey or Turkmenistan or Turkmen or Uganda or Ukraine or Uruguay or USSR or Soviet Union or Union of Soviet Socialist Republics or Uzbekistan or Uzbek or Vanuatu or New Hebrides or Venezuela or Vietnam or Viet Nam or West Bank or Yemen or Yugoslavia or Zambia or Zimbabwe or Rhodesia).hw,kf,ti,ab,cp
4. ((developing or less* developed or under developed or underdeveloped or middle income or low* income or underserved or under served or deprived or poor*) adj (countr* or nation? or population? or world)).ti,ab
5. ((developing or less* developed or under developed or underdeveloped or middle income or low* income) adj (economy or economies)).ti,ab
6. (low* adj (gdp or gnp or gross domestic or gross national)).ti,ab
7. (low adj3 middle adj3 countr*).ti,ab.
8. (lmic or lmics or third world or lami countr*).ti,ab.
9. transitional countr*.ti,ab.
10. Or/64-72 (4137)

***D: Controlled study***

1. randomized controlled trial.pt
2. controlled clinical trial.pt.
3. multicenter study.pt.
4. (randomised or randomized or randomly).ti,ab
5. placebo.ti,ab
6. trial.ti,ab
7. groups.ti,ab.
8. intervention.ti,ab.
9. evaluat*.ti,ab
10. control*.ti,ab.
11. effect?.ti,ab.
12. impact.ti,ab
13. (time series or time points).ti,ab.
14. ((pretest or pre test) and (posttest or post test)).ti,ab.
15. (quasi experiment* or quasiexperiment*).ti,ab
16. ((multicentre or multicentre or multi centre or multi center) adj study).ti,ab.
17. Or/ 74-89
18. 5 and 63 and 73 and 90
