# Supplementary material for: Community-based psychosocial interventions for people with schizophrenia in low and middle-income countries: systematic review and meta-analysis
Source: BMC Psychiatry. 2017 Oct 30;17:355. doi: 10.1186/s12888-017-1516-7 (PMC5661919; doi:10.1186/s12888-017-1516-7)
Supplement: Supplementary file 3 — Reasons for exclusion of full texts. (DOCX 202 kb) [file 12888_2017_1516_MOESM3_ESM.docx]

## Reasons for exclusion of full texts

| **Reference** | **Reason for exclusion** |
| --- | --- |
| Arslan 2014 [1] | Non-eligible intervention (health facility only) |
| Bio 2011 [2] | Non-eligible intervention (work placement only) |
| Barretto 2009 [3] | Non-eligible intervention (health facility only) |
| Dikec 2016 [4] | Non-eligible intervention (health facility only) |
| Farooq 2011 [5] | Non-eligible intervention (health facility only) |
| Gohar 2013 [6] | Non-eligible intervention (health facility only) |
| Guo 2010 [7] | Non-eligible intervention (health facility only) |
| Hasan 2015 [8] | Non-eligible intervention (telephone contact / health facility only) |
| Idaiani 2015 [9] | Non-eligible methodology (historical/ retrospective cohort) |
| Kooalee 2010 [10] | Non-eligible intervention (health facility only) |
| Kulhara 2009 [11] | Non-eligible intervention (health facility only) |
| Kumar 2008 [12] | Non-eligible intervention (health facility only) |
| Li 2015 [13] | Non-eligible intervention (health facility only) |
| Maneesakorn 2007 [14] | Non-eligible intervention (health facility only) |
| Naeem 2015 [15] | Non-eligible intervention (health facility only) |
| Padma Sari 2014 [16] | Non-eligible intervention (health facility only) |
| Pan 2011 [17] | Non-eligible intervention (health facility only) |
| Paranthaman 2010 [18] | Non-eligible intervention (health facility only) |
| Pontes 2013 [19] | Non-eligible intervention (health facility only) |
| Prost 2013 [20] | Non-eligible intervention (health facility only) |
| Razali 2015 [21] | Non-eligible methodology (historical/ retrospective cohort) |
| Sharif 2012 [22] | Non-eligible intervention (health facility only) |
| Tao 2012 [23] | Non-eligible intervention (health facility only- day centre) |
| Tas 2012 [24] | Non-eligible intervention (health facility only) |
| Valencia 2013 [25] | Non-eligible intervention (health facility only) |
| Valencia 2012 [26] | Non-eligible intervention (health facility only) |
| Valencia 2007 [27] | Non-eligible intervention (health facility only) |
| Valencia 2010 [28] | Non-eligible intervention (health facility only) |
| Wang 2013a [29] | Non-eligible intervention (health facility only) |
| Wang 2013b [30] | Non-eligible intervention (health facility only) |
| Xiang 2006 [31] | Non-eligible intervention (health facility only) |
| Xiang 2007 [32] | Non-eligible intervention (health facility only) |
| Xiong 1994 [33] | Non-eligible intervention (health facility only) |
| Yildiz 2004 [34] | Non-eligible intervention (health facility only) |
| Yildirim 2015 [35] | Non-eligible intervention (health facility only) |
| Zhang 1993 [36] | Non-eligible intervention (health facility only) |
| Zhang 1998 [37] | Non-eligible intervention (health facility only) |
| Zhang 2014 [38] | Non-eligible intervention (health facility only) |
| Zhou 2014 [39] | Non-eligible intervention (health facility only) |
| Zhou 2015 [40] | Non-eligible intervention (health facility only- day centre) |
| Zimmer 2007 [41] | Non-eligible intervention (health facility only) |

1. Arslan M, Kurt E, Eryildiz D, Yazici A, Can A, Emul M: **Effects of a psychosocial rehabilitation program in addition to medication in schizophrenic patients: A controlled study**. *Klinik Psikofarmakoloji Bulteni* 2014, **24**(4):360-367.

2. Bio DS, Gattaz WF: **Vocational rehabilitation improves cognition and negative symptoms in schizophrenia**. *Schizophr Res* 2011, **126**(1-3):265-269.

3. de Paiva Barretto EM, Kayo M, Avrichir BS, Sa AR, Camargolutti MdGM, Napolitano IC, Nery FG, Pinto JA, Jr., Bannwart S, Scemes S *et al*: **A preliminary controlled trial of cognitive behavioral therapy in clozapine-resistant schizophrenia**. *Journal of Nervous and Mental Disease* 2009, **197**(11):865-868.

4. Dikec G, Kutlu Y: **Effectiveness of Adherence Therapy for People With Schizophrenia in Turkey: A Controlled Study**. *Arch Psychiatr Nurs* 2016, **30**(2):249-256 248p.

5. Farooq S, Nazar Z, Irfan M, Akhter J, Gul E, Irfan U, Naeem F: **Schizophrenia medication adherence in a resource-poor setting: randomised controlled trial of supervised treatment in out-patients for schizophrenia (STOPS)**. *The British Journal of Psychiatry* 2011, **199**(6):467-472.

6. Gohar SM, Hamdi E, El Ray LA, Horan WP, Green MF: **Adapting and evaluating a social cognitive remediation program for schizophrenia in Arabic**. *Schizophr Res* 2013, **148**(1-3):12-17.

7. Guo X, Zhai J, Liu Z, Fang M, Wang B, Wang C, Hu B, Sun X, Lv L, Lu Z *et al*: **Effect of antipsychotic medication alone vs combined with psychosocial intervention on outcomes of early-stage schizophrenia: A randomized, 1-year study**. *Arch Gen Psychiatry* 2010, **67**(9):895-904.

8. Hasan AA, Callaghan P, Lymn JS: **Evaluation of the impact of a psycho-educational intervention for people diagnosed with schizophrenia and their primary caregivers in Jordan: A randomized controlled trial**. *BMC Psychiatry Vol 15 Apr 2015, ArtID 72* 2015, **15**.

9. Idaiani S: **The effectiveness of community-based mental health program by community health centers on the recovery of patients with psychosis in Aceh**. *ASEAN Journal of Psychiatry* 2015, **16**(2):212-221.

10. Koolaee AK, Etemadi A: **The outcome of family interventions for the mothers of schizophrenia patients in Iran**. *Int J Soc Psychiatry* 2010, **56**(6):634-646.

11. Kulhara P, Chakrabarti S, Avasthi A, Sharma A, Sharma S: **Psychoeducational intervention for caregivers of Indian patients with schizophrenia: a randomised-controlled trial**. *Acta Psychiatr Scand* 2009, **119**(6):472-483.

12. Kumar PNS: **Impact of vocational rehabilitation on social functioning, cognitive functioning, and psychopathology in patients with chronic schizophrenia**. *Indian Journal of Psychiatry* 2008, **50**(4):257-261 255p.

13. Li ZJ, Guo ZH, Wang N, Xu ZY, Qu Y, Wang XQ, Sun J, Yan LQ, Ng RMK, Turkington D *et al*: **Cognitive–behavioural therapy for patients with schizophrenia: a multicentre randomized controlled trial in Beijing, China**. *Psychol Med* 2015, **45**(9):1893-1905 1813p.

14. Maneesakorn S, Robson D, Gournay K, Gray R: **An RCT of adherence therapy for people with schizophrenia in Chiang Mai, Thailand**. *J Clin Nurs* 2007, **16**(7):1302-1312.

15. Naeem F, Saeed S, Irfan M, Kiran T, Mehmood N, Gul M, Munshi T, Ahmad S, Kazmi A, Husain N *et al*: **Brief culturally adapted CBT for psychosis (CaCBTp): A randomized controlled trial from a low income country**. *Schizophr Res* 2015, **164**(1-3):143-148.

16. Padma Sari S, Suttharangsee W, Chanchong W, Turale S: **Self-Management Family Participation Program for Medication Adherence among Indonesian People with Schizophrenia: A Randomised Controlled Trial Study**. *Pacific Rim International Journal of Nursing Research* 2014, **18**(4):274-289 216p.

17. Pan L, Mellor D, McCabe M, Hill B, Tan W, Xu Y: **An evaluation of the shanghai mental health service schizophrenia rehabilitation program**. *American Journal of Psychiatric Rehabilitation* 2011, **14**(4):287-306.

18. Paranthaman V, Satnam K, Lim JL, Amar-Singh HSS, Sararaks S, Nafiza MN, Ranjit K, Asmah ZA: **Effective implementation of a structured psychoeducation programme among caregivers of patients with schizophrenia in the community**. *Asian J Psychiatr* 2010, **3**(4):206-212.

19. Pontes LM, Martins CB, Napolitano IC, Fonseca JR, Oliveira GM, Iso SM, Menezes AK, Vizzotto AD, di Sarno ES, Elkis H: **Cognitive training for schizophrenia in developing countries: a pilot trial in Brazil**. *Schizophr Res Treatment* 2013, **2013**:321725.

20. Prost E, Musisi S, Okello ES, Hopman WM: **The role of psycho-education in improving outcome at a general hospital psychiatry clinic in Uganda**. *African Journal of Psychiatry (South Africa)* 2013, **16**(4):264-270.

21. Razali S, Hashim M: **Modified Assertive Community Treatment: Effectiveness on Hospitalization and Length of Stay**. *Community Ment Health J* 2015, **51**(2):171-174 174p.

22. Sharif F, Shaygan M, Mani A: **Effect of a psycho-educational intervention for family members on caregiver burdens and psychiatric symptoms in patients with schizophrenia in Shiraz, Iran**. *BMC Psychiatry Vol 12 May 2012, ArtID 48* 2012, **12**.

23. Tao H, Song L, Niu X, Li X, Zhang Q, Cui J, Chen H, Fu Z, Fang W: **Effectiveness of a rehabilitative program that integrates hospital and community services for patients with schizophrenia in one community in Shanghai**. *Shanghai Archives of Psychiatry* 2012, **24**(3):140-148.

24. Tas C, Danaci AE, Cubukcuoglu Z, Brune M: **Impact of family involvement on social cognition training in clinically stable outpatients with schizophrenia -- a randomized pilot study**. *Psychiatry Res* 2012, **195**(1-2):32-38.

25. Valencia M, Fresan A, Juarez F, Escamilla R, Saracco R: **The beneficial effects of combining pharmacological and psychosocial treatment on remission and functional outcome in outpatients with schizophrenia**. *J Psychiatr Res* 2013, **47**(12):1886-1892.

26. Valencia M, Juarez F, Ortega H: **Integrated treatment to achieve functional recovery for first-episode psychosis**. *Schizophrenia research and treatment* 2012, **2012**:962371-962371.

27. Valencia M, Rascon ML, Juarez F, Murow E: **A psychosocial skills training approach in Mexican out-patients with schizophrenia**. *Psychol Med* 2007, **37**(10):1393-1402 1310p.

28. Valencia M, Rascon ML, Juarez F, Escamilla R, Saracco R, Liberman RP: **Application in Mexico of psychosocial rehabilitation with schizophrenia patients**. *Psychiatry: Interpersonal and Biological Processes* 2010, **73**(3):248-263.

29. Wang L, Zhou J, Yu X, Qiu J, Wang B: **Psychosocial rehabilitation training in the treatment of schizophrenia outpatients: A randomized, psychosocial rehabilitation training-and monomedication-controlled study**. *Pakistan Journal of Medical Sciences* 2013, **29**(2).

30. Wang Y, Roberts DL, Xu B, Cao R, Yan M, Jiang Q: **Social cognition and interaction training for patients with stable schizophrenia in Chinese community settings**. *Psychiatry Res* 2013, **210**(3):751-755.

31. Xiang Y, Weng Y, Li W, Gao L, Chen G, Xie L, Chang Y, Tang WK, Ungvari GS: **Training patients with schizophrenia with the community re-entry module: A controlled study**. *Social Psychiatry and Psychiatric Epidemiology* 2006, **41**(6):464-469.

32. Xiang Y-T, Weng Y-Z, Li W-Y, Gao L, Chen G-L, Xie L, Chang Y-L, Tang W-K, Ungvari GS: **Efficacy of the Community Re-Entry Module for patients with schizophrenia in Beijing, China: outcome at 2-year follow-up**. *The British Journal of Psychiatry* 2007, **190**(1):49-56.

33. Xiong W, Phillips MR, Hu X, Wang R, Dai Q, Kleinman J, Kleinman A: **Family-based intervention for schizophrenic patients in China: A randomised controlled trial**. *The British Journal of Psychiatry* 1994, **165**(2):239-247.

34. Yildiz M, Veznedaroglu B, Eryavuz A, Kayahan B: **Psychosocial skills training on social functioning and quality of life in the treatment of schizophrenia: A controlled study in Turkey**. *Int* 2004, **8**(4):219-225.

35. Yıldırım A, Aşılar RH, Camcıoğlu TH, Erdiman S, Karaağaç E: **Effect of Psychosocial Skills Training on Disease Symptoms, Insight, Internalized Stigmatization, and Social Functioning in Patients with Schizophrenia**. *Rehabilitation Nursing* 2015, **40**(6):341-348 348p.

36. Zhang M, Yan H, Yao C, Ye J, et al.: **Effectiveness of psychoeducation of relatives of schizophrenic patients: A prospective cohort study in five cities of China**. *International Journal of Mental Health* 1993, **22**(1):47-59.

37. Zhang MY, Zhu ZQ, Yan HQ, Song CC, Liu FZ: **Group psychosocial education for relatives of schizophrenic patients in community: A three-year experience**. *Hong Kong Journal of Psychiatry* 1998, **8**(1):33-37.

38. Zhang Z, Zhai J, Wei Q, Qi J, Guo X, Zhao J: **Cost-effectiveness analysis of psychosocial intervention for early stage schizophrenia in China: A randomized, one-year study**. *BMC Psychiatry Vol 14 Jul 2014, ArtID 212* 2014, **14**.

39. Zhou B, Zhang P, Gu Y: **Effectiveness of self-management training in community residents with chronic schizophrenia: A single-blind randomized controlled trial in Shanghai, China**. *Shanghai Archives of Psychiatry* 2014, **26**(2):81-87.

40. Zhou Y, Zhou R, Li W, Lin Y, Yao J, Chen J, Shen T: **Controlled trial of the effectiveness of community rehabilitation for patients with schizophrenia in Shanghai, China**. *Shanghai Archives of Psychiatry* 2015, **27**(3):167-174.

41. Zimmer M, Duncan AV, Laitano D, Ferreira EE, Belmonte-de-Abreu P: **A twelve-week randomized controlled study of the cognitive-behavioral Integrated Psychological Therapy program: Positive effect on the social functioning of schizophrenic patients**. *Rev Bras Psiquiatr* 2007, **29**(2):140-147.
