# Supplementary material for: Community-based psychosocial interventions for people with schizophrenia in low and middle-income countries: systematic review and meta-analysis
Source: BMC Psychiatry. 2017 Oct 30;17:355. doi: 10.1186/s12888-017-1516-7 (PMC5661919; doi:10.1186/s12888-017-1516-7)
Supplement: Supplementary file 4 — Summary of risk of bias for included studies. (DOCX 132 kb) [file 12888_2017_1516_MOESM4_ESM.docx]

# Additional file 4: Summary of risk of bias in included studies

|  | **Random sequence generation (selection bias)** | **Allocation concealment (selection bias)** | **Blinding of outcome assessment (detection bias)** | **Incomplete outcome data (attrition bias)** | **Selective reporting (reporting bias)** | **Other bias** | **Overall risk of bias** |
| --- | --- | --- | --- | --- | --- | --- | --- |
| **Botha 2014** | 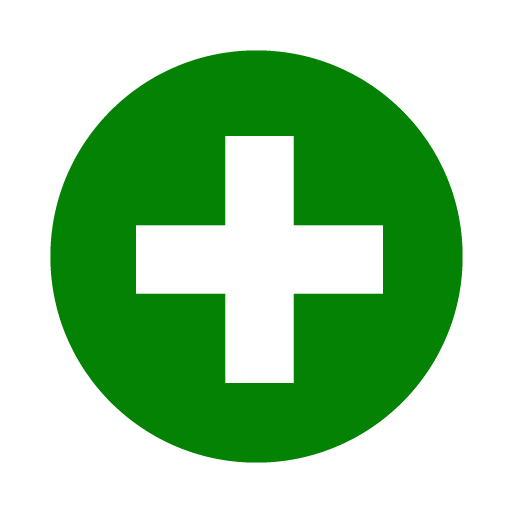 | 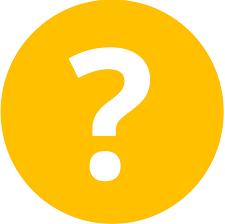 | 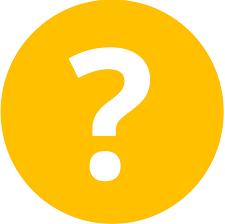 | 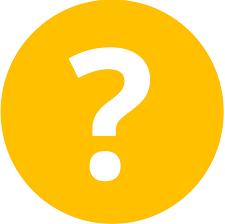 | 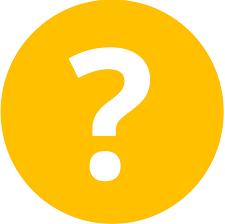 | 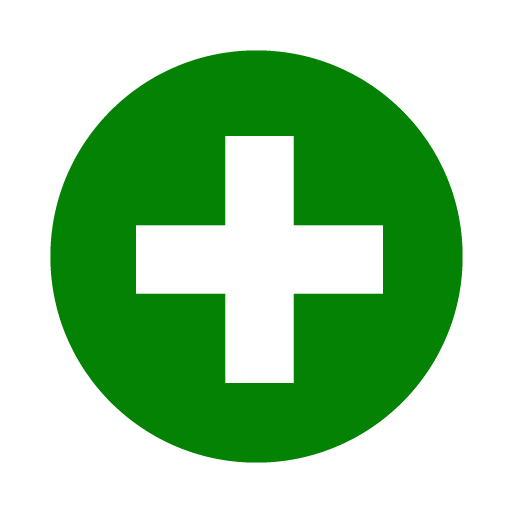 | Low/ unclear |
| **Cai 2015** | 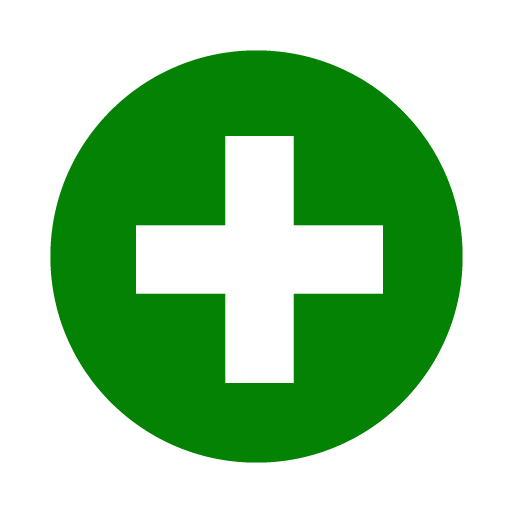 | 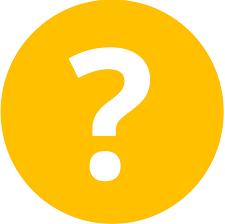 | 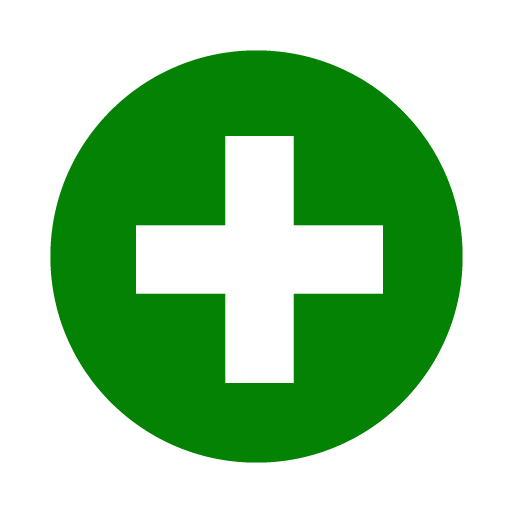 | 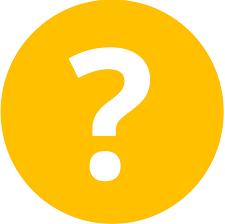 | 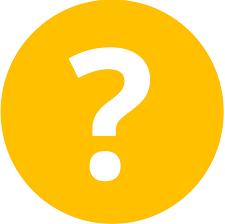 | 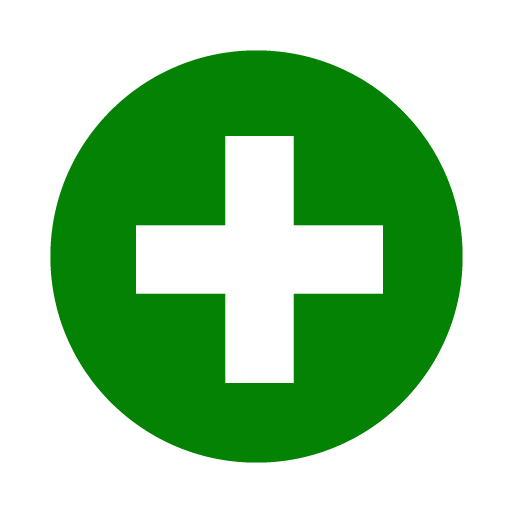 | Low/ unclear |
| **Chatterjee 2014** | 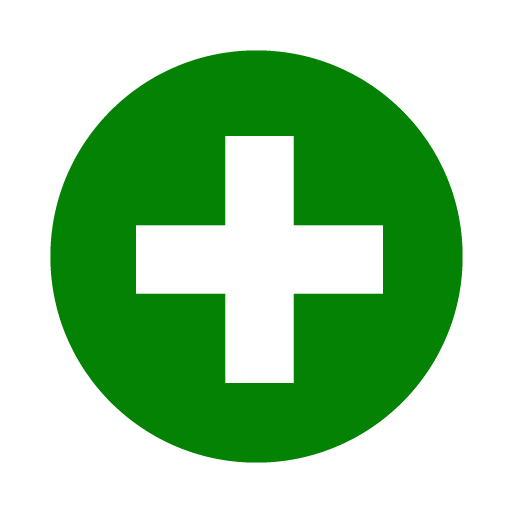 | 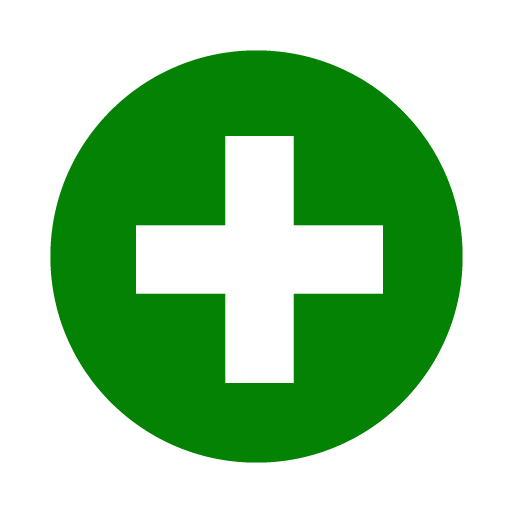 | 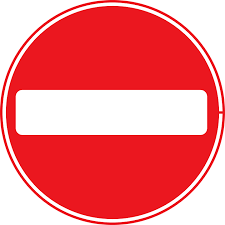 | 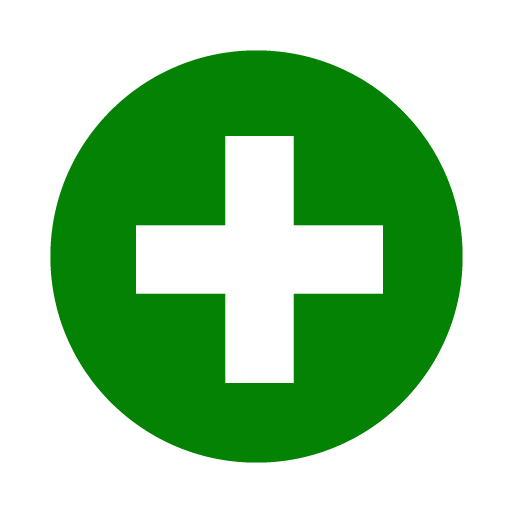 | 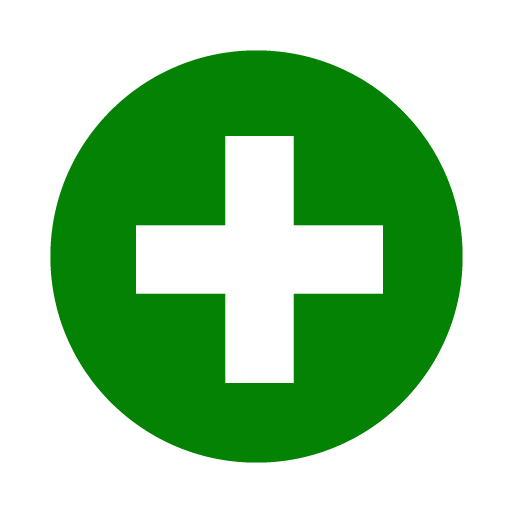 | 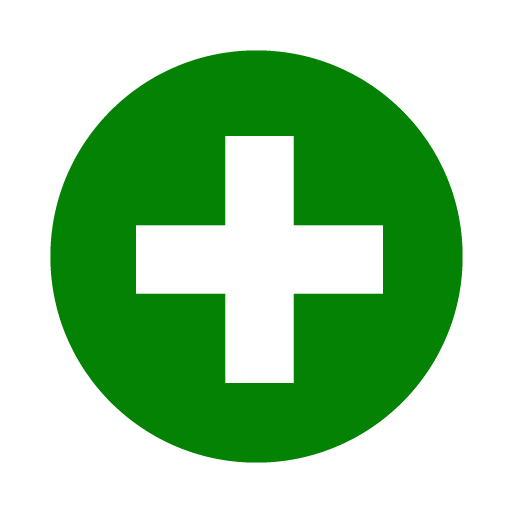 | Low |
| **Ghadiri 2015** | 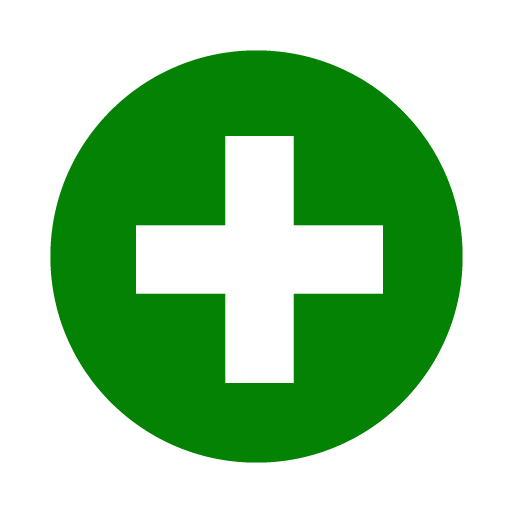 | 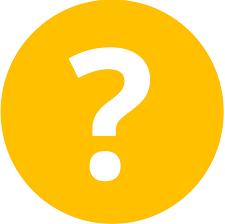 | 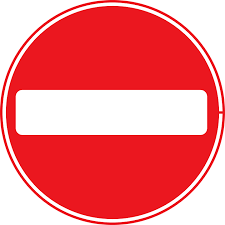 | 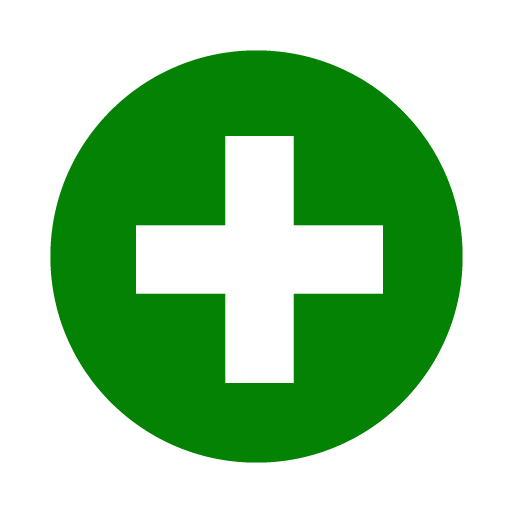 | 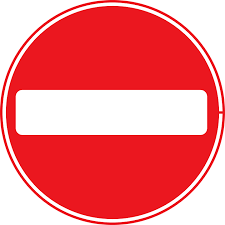 | 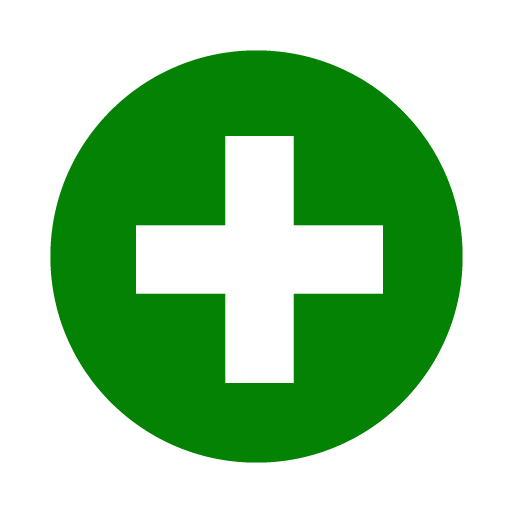 | High |
| **Hegde 2012** | 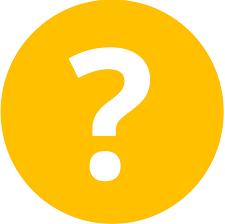 | 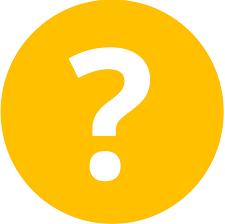 | 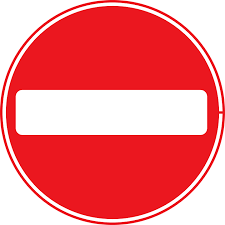 | 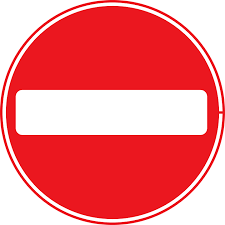 | 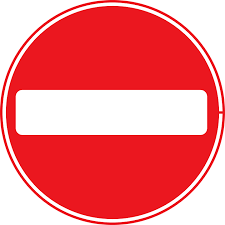 | 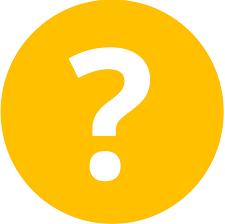 | High |
| **Li 2005** | 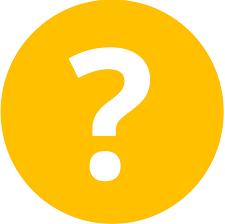 | 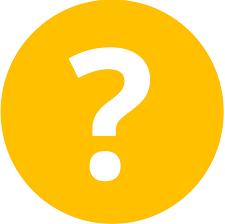 | 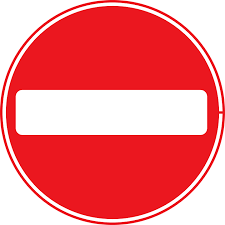 | 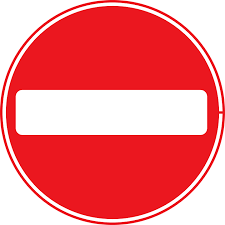 | 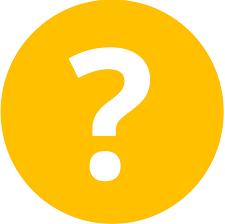 | 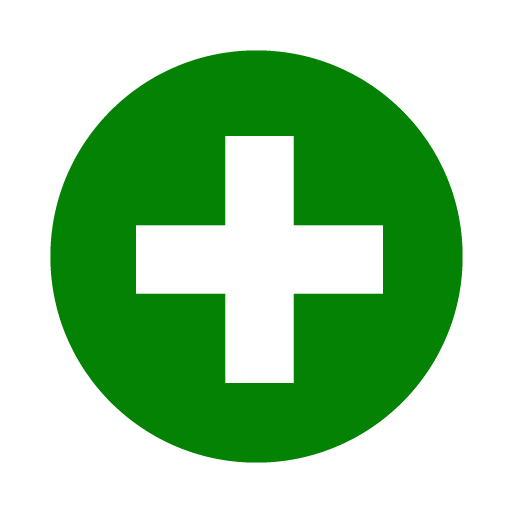 | High |
| **Ran 2015** | 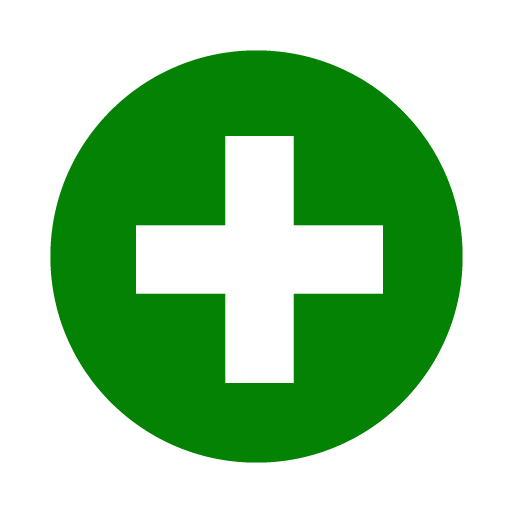 | 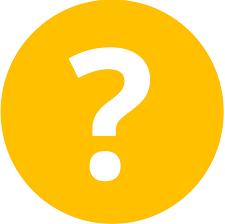 | 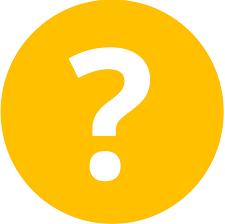 | 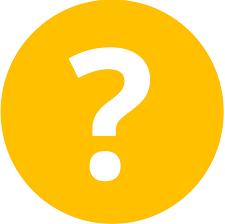 | 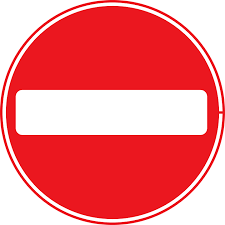 | 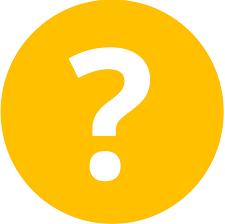 | Low/ unclear |
| **Sharifi 2012** | 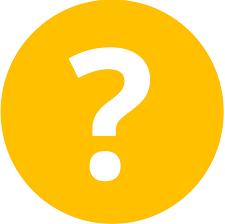 | 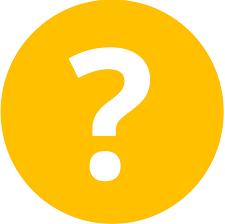 | 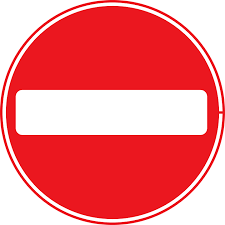 | 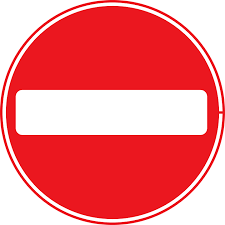 | 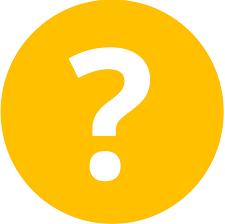 | 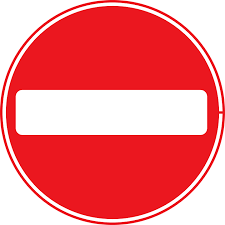 | High |
| **Sungur 2011** | 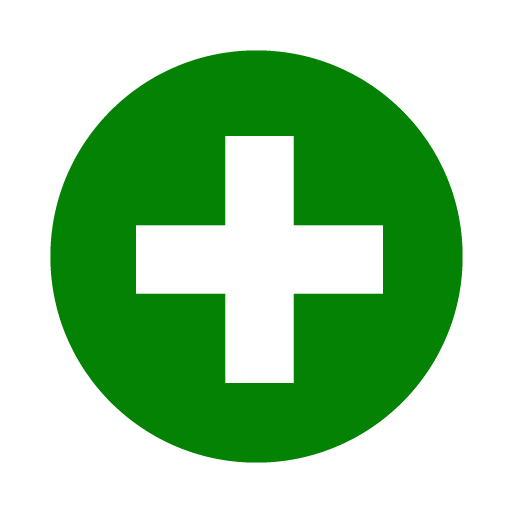 | 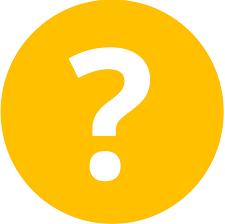 | 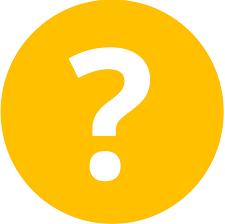 | 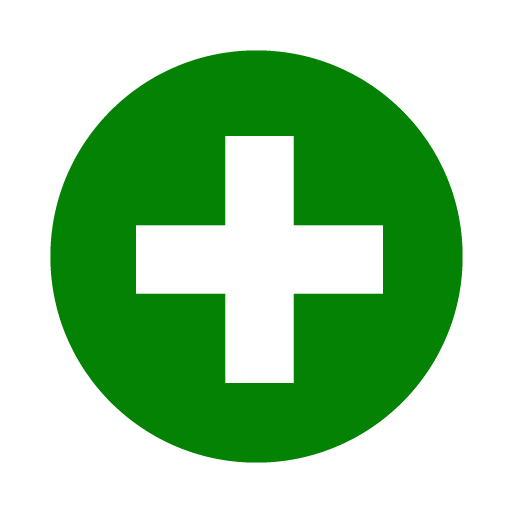 | 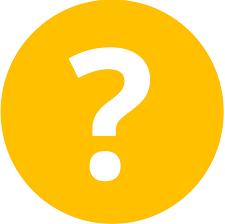 | 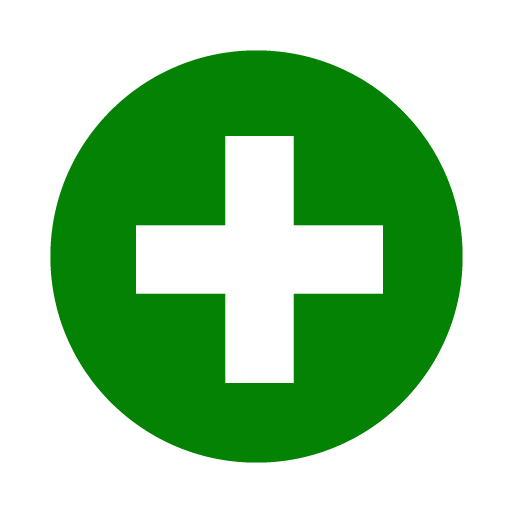 | Low/ unclear |
| **Xiang 1994** | 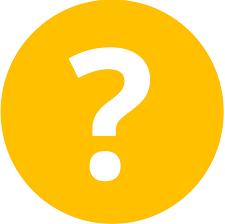 | 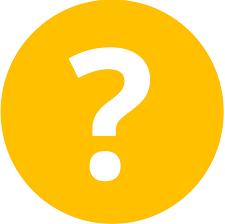 | 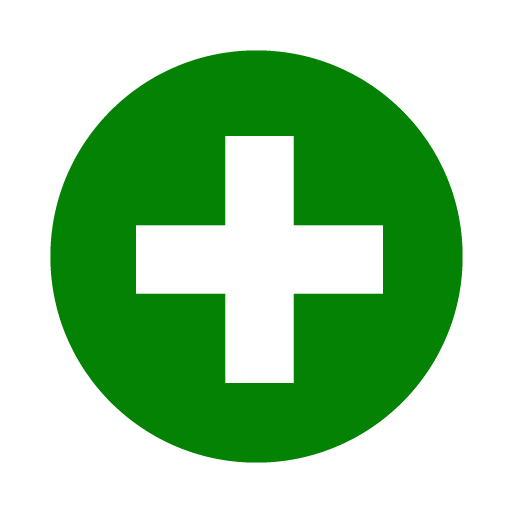 | 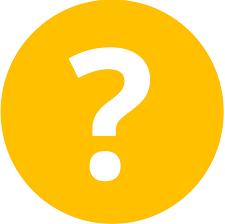 | 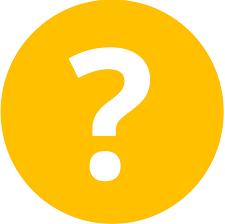 | 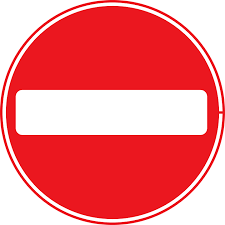 | Low/ unclear |
| **Zhang 1994** | 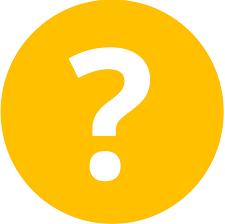 | 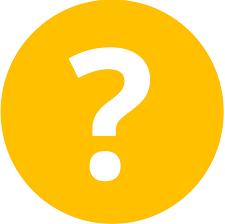 | 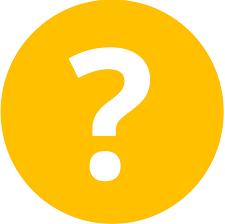 | 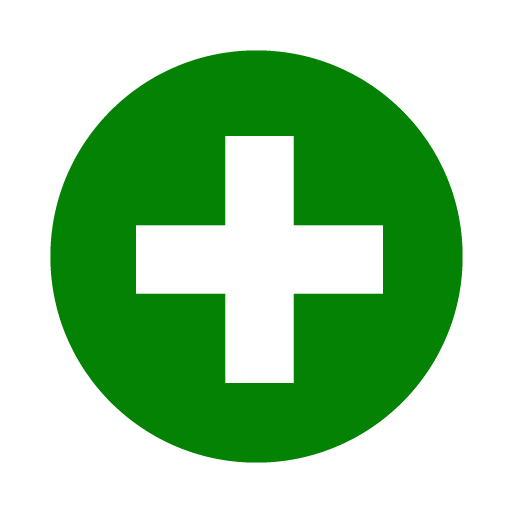 | 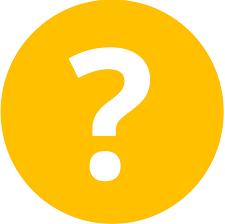 | 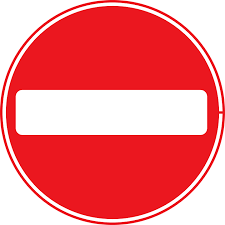 | Low/ unclear |
