# Supplementary material for: Community-based psychosocial interventions for people with schizophrenia in low and middle-income countries: systematic review and meta-analysis
Source: BMC Psychiatry. 2017 Oct 30;17:355. doi: 10.1186/s12888-017-1516-7 (PMC5661919; doi:10.1186/s12888-017-1516-7)
Supplement: Supplementary file 5 — Additional analyses. This file presents the forest plots for symptom severity (<18 months post intervention) including only high-quality studies; impact on ability to work (<18 months post intervention) including all studies; impact on number of readmissions (<18 months post intervention) including all studies; impact on number of days in hospital (<18 months post intervention); and medication adherence (<18 months post intervention) including only high-quality studies. (DOCX 254 kb) [file 12888_2017_1516_MOESM5_ESM.docx]

## Additional file 5: Additional analyses


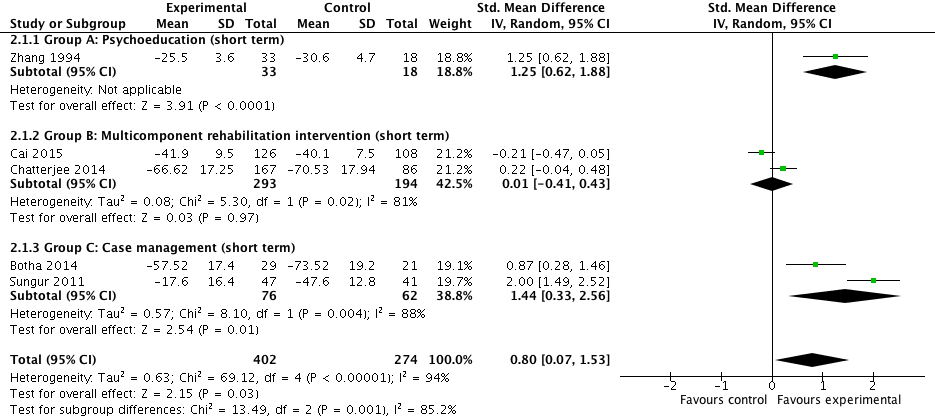


Figure a: Community-based psychosocial intervention versus usual care: impact on symptom severity (<18 months post intervention and high quality studies)


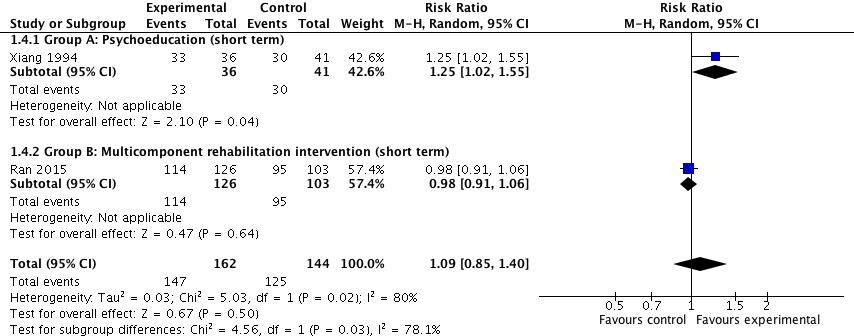


Figure b: Community-based psychosocial intervention versus usual care: impact on ability to work (<18 months post intervention)


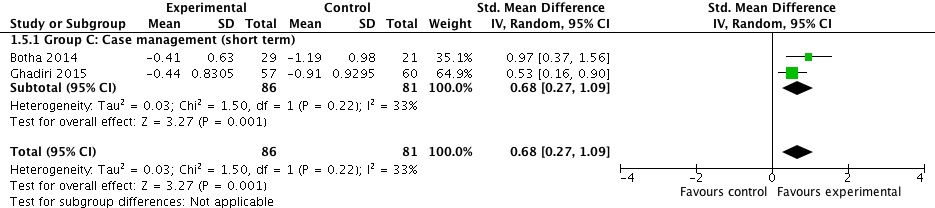


Figure c: Community-based psychosocial intervention versus usual care: impact on number of readmissions (<18 months post intervention)


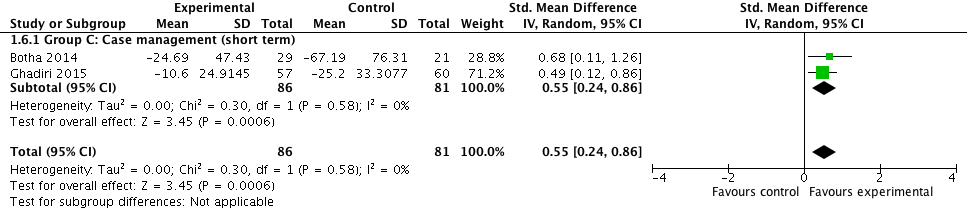


Figure d: Community-based psychosocial interventions versus usual care: impact on number of days in hospital (<18 months post intervention)


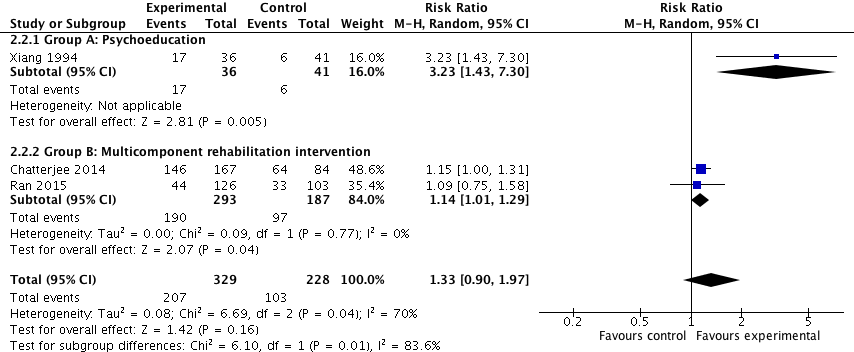


Figure e: Community-based psychosocial intervention versus usual care: impact on medication adherence (<18 months post intervention and high quality studies)
