# Supplementary material for: Community-based psychosocial interventions for people with schizophrenia in low and middle-income countries: systematic review and meta-analysis
Source: BMC Psychiatry. 2017 Oct 30;17:355. doi: 10.1186/s12888-017-1516-7 (PMC5661919; doi:10.1186/s12888-017-1516-7)
Supplement: Supplementary file 6 — Funnel plot for symptom severity. (PDF 65 kb) [file 12888_2017_1516_MOESM6_ESM.pdf]

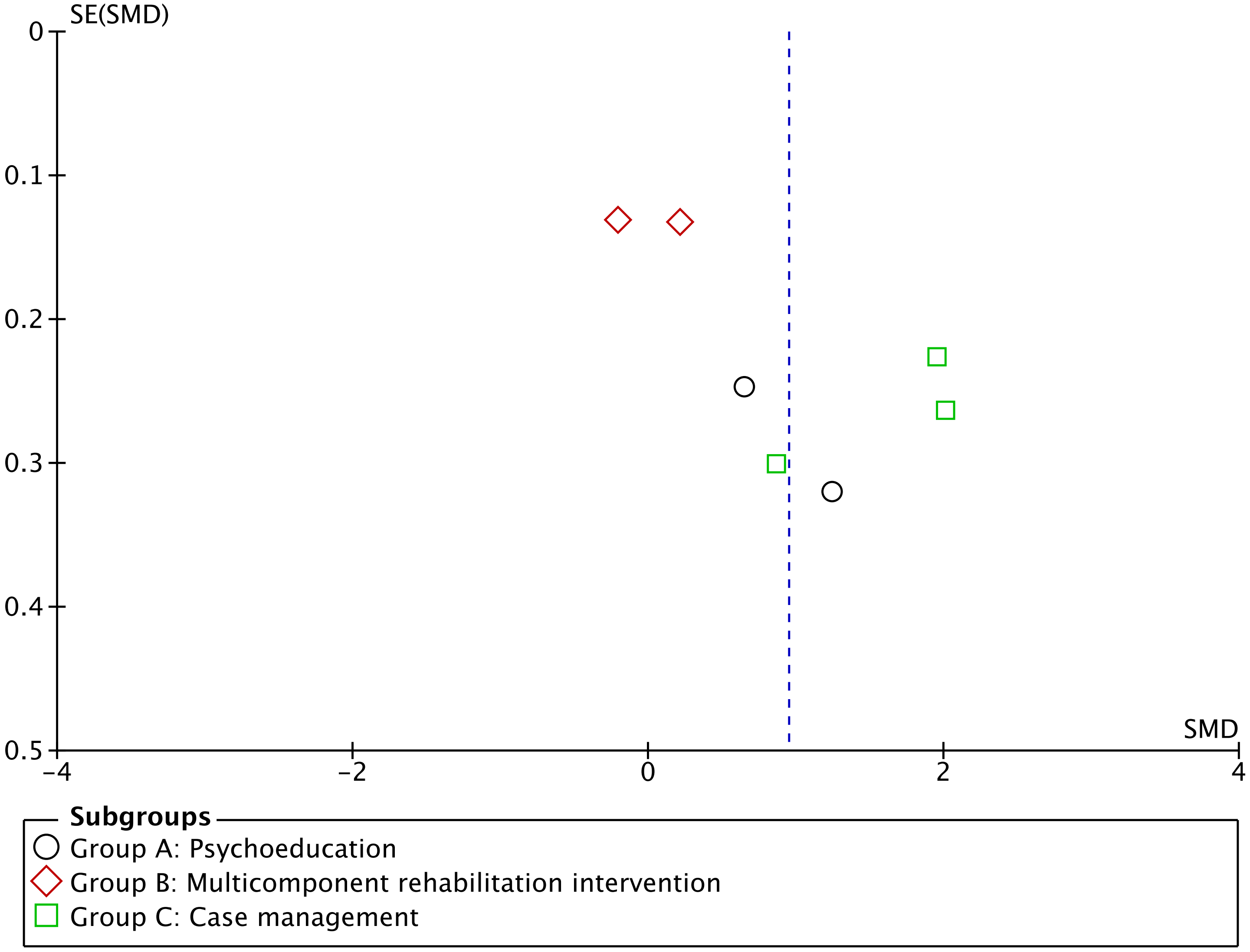

SE(SMD)
0
0.1
0.2
0.3
0.4
0.5
SMD
−4
−2
0
2
4
Subgroups
Group A: Psychoeducation
Group B: Multicomponent rehabilitation intervention
Group C: Case management
